# A Correlation-Based Approach for Predicting Humic Substance Bioactivity from Direct Compost Characterization

**DOI:** 10.3390/molecules30071511

**Published:** 2025-03-28

**Authors:** Ana Catarina Silva, Pedro Rocha, Patrícia Valderrama, Juan Antelo, Dulce Geraldo, Maria Fernanda Proença, Sarah Fiol, Fátima Bento

**Affiliations:** 1 Department of Chemistry, Centre of Chemistry, University of Minho, Campus Gualtar, 4710-057 Braga, Portugal; a.catarinasilva@2c2t.uminho.pt (A.C.S.); jprocha@quimica.uminho.pt (P.R.); gdulce@quimica.uminho.pt (D.G.); fproenca@quimica.uminho.pt (M.F.P.); 2 CRETUS, Cross-Disciplinary Research in Environmental Technologies, Department of Physical Chemistry, University of Santiago de Compostela, 15782 Santiago de Compostela, Spain; sarah.fiol@usc.es; 3 Campus Campo Mourão (UTFPR-CM), Universidade Tecnológica Federal do Paraná, Campo Mourão 87301-899, Paraná, Brazil; pativalderrama@gmail.com; 4 CRETUS, Cross-Disciplinary Research in Environmental Technologies, Department of Soil Science and Agricultural Chemistry, University of Santiago de Compostela, 15782 Santiago de Compostela, Spain; juan.antelo@usc.es

**Keywords:** compost characterization, humic-like substances, correlation analysis, compost maturity, bioactivity, sustainable agriculture, thermal stability, compost quality index

## Abstract

The efficient characterization of compost quality is essential for optimizing its application in agriculture and soil improvement. In this study, a correlation-based approach was employed to evaluate relationships between physicochemical properties, structural features, and reactivity indicators of compost extracts—fulvic acid-like (FA-L), humic acid-like (HA-L), and dissolved organic matter (DOM)—and their respective bulk composts. The goal was to identify key compost parameters that can serve as reliable predictors of humic substance composition and bioactivity, thereby reducing reliance on labor-intensive humic substance extractions. A comprehensive set of elemental, spectroscopic (UV-vis, ATR-FTIR, ^1^H-NMR), and thermal (TGA-DSC) analyses were conducted to assess the composition and stability of the extracts. Strong correlations were found between compost oxidation state (*C_oxi_*/*C*), cation exchange capacity (*CEC*), thermal stability, and the structural characteristics of humic substances-like (HS-L) fractions, suggesting that direct compost characterization can effectively predict humic substance reactivity and agronomic potential. The findings also align with a previously developed Compost Quality Index (CQI), reinforcing the functional role of humic substances in soil fertility and nutrient retention. By establishing a simplified yet robust compost assessment framework, this study advances the potential for efficient, cost-effective evaluation methodologies for compost quality.

## 1. Introduction

The sustainable management of organic waste is a growing global challenge, with composting emerging as a key strategy to recycle organic biowaste into valuable soil amendments. Compost plays a crucial role in enhancing soil fertility, increasing organic matter content, and improving nutrient retention, making it an essential component of sustainable agriculture and circular economy initiatives [1]. The beneficial properties of compost are largely attributed to its organic fraction, particularly the humic-like substances (HS-L), which include fulvic acid-like (FA-L) and humic acid-like (HA-L) fractions. These substances modulate soil structure, enhance cation exchange capacity (CEC), facilitate metal complexation, and promote microbial activity, directly influencing soil health and crop productivity [2].

While the presence of HS-L in compost is widely recognized as a quality indicator, their quantification and characterization remain analytically challenging. The conventional assessments require multi-step chemical extractions, followed by characterization by spectroscopic (UV-vis, ATR-FTIR, ^1^H-NMR), thermal (TGA-DSC), and electrochemical methods, to evaluate aromaticity, functional groups, and reactivity [3]. Although these methodologies provide valuable insights into compost maturity and stability, they are time-consuming and labor-intensive.

Several studies have attempted to identify proxy indicators for compost maturity and humic substance bioactivity. Spectroscopic techniques such as UV-vis and ATR-FTIR have been used to track changes in aromaticity, oxidation, and functional group composition during composting [4,5,6]. Thermal analysis methods (TGA-DSC) have been employed to assess the stability and recalcitrance of organic matter, distinguishing between labile and persistent carbon fractions [6]. Electrochemical techniques have recently gained attention for their ability to quantify the reactivity of HS-L and their interactions with metal ions [7,8]. Despite these advances, the challenge remains: Can compost quality be reliably assessed without labor-intensive humic substance extractions?

In our previous study we proposed a Compost Quality Index (CQI), integrating electrochemical quantification of Cd^2+^ binding to HS-L to assess compost bioactivity [9]. The CQI was validated using a lettuce bioassay, demonstrating that composts with highly reactive HS-L fractions exhibited enhanced plant growth. However, while the CQI provided an efficient quality indicator system, it still required HS-L extractions and electrochemical measurements, limiting its feasibility for routine analysis. This raised a crucial question: Can bulk compost properties serve as reliable predictors of HS-L bioactivity, eliminating the need for extraction-based characterization?

In environmental chemistry, correlation-based analyses have been widely employed to establish predictive relationships between complex chemical systems, particularly in soil and water quality assessments [10,11]. By identifying statistically significant associations between measurable parameters, correlation-based models allow for the development of surrogate indicators that can simplify routine analyses [12,13,14]. Such approaches have been successfully applied in soil organic matter stability studies, where oxidation indices and thermal degradation parameters have been used to infer microbial decomposition potential [15]. However, their application to compost quality assessment and HS-L bioactivity prediction remains largely unexplored.

To address this gap, this study applies a correlation-based approach to predict HS-L bioactivity from compost chemical characterization. By analyzing a diverse set of compost samples produced from different feedstocks (Table S1) and composting methods (industrial composting, home composting, and vermicomposting), we systematically evaluate whether bulk physicochemical properties, such as oxidation state, elemental composition, CEC, and thermal stability, can serve as reliable indicators of HS-L characteristics.

To achieve this main goal, in this work we have established relationships between compost features and HS-L composition and reactivity that are correlated with its bioactivity. Thus, this study contributes to the ongoing effort to develop more efficient and cost-effective methodologies for compost characterization, addressing the need for simplified but scientifically robust assessments of compost maturity and bioactivity. If successful, this approach could significantly simplify compost characterization protocols, making them more accessible for routine use in composting facilities, agricultural applications, and environmental monitoring. By using correlation-based analysis, this work provides the base for future studies that integrate machine learning and predictive modeling into compost quality assessment, further enhancing the efficiency of sustainable soil management practices.

## 2. Results and Discussion

### 2.1. Physico-Chemical and Spectroscopic Characterization of Compost Extracts

#### 2.1.1. Dissolved Organic Matter (DOM) Extract

- Physico-chemical characterization

The physico-chemical properties of the dissolved organic matter (DOM) extracts obtained in equilibrium solutions from composts and fertilizer are summarized in Table S2. Dissolved organic carbon (DOC) concentrations ranged from 111 to 861 mg dm^−3^, with the highest value observed in the compost of livestock waste (CLW). Electrical conductivity (EC), which reflects the salinity of the extracts, exceeded the 4 S cm^−1^ threshold for agricultural applications [16] only in CLW, whereas the remaining samples exhibited relatively low EC values.

The pH values varied between 6.3 and 8.5, with the highest value recorded for CLW and the lowest for the vermicompost samples, vermicompost of algae (CVA), and vermicompost of domestic waste (CVDW). These results suggest that vermicomposting tends to produce more acidic extracts compared to traditional composting, particularly when using the same feedstock. This trend is evident when comparing domestic compost of domestic waste (CDDW) and vermicompost of domestic waste (CVDW), where the latter has a lower pH.

**Figure 1:**
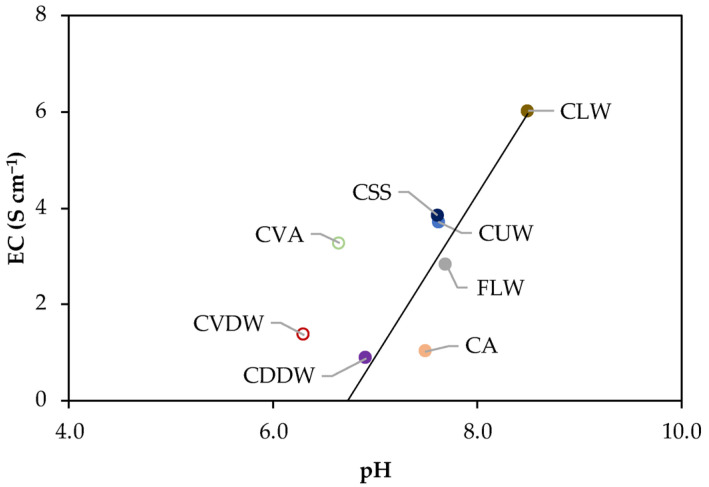
Correlation of the electric conductivity (EC) as a function of the pH of the DOM extracts of the compost samples, where a full line represents the linear fitting (*r* = 0.89). Data from CVA and CVDW (open symbols) were not included in the fitting.

A strong positive correlation between pH and electrical conductivity (EC) (*r* = 0.89) was observed (Figure 1), suggesting that cation adsorption onto pH-dependent acidic functional groups in organic matter plays a key role in regulating EC. However, the extent of this relationship differed between composted and vermicomposted samples, indicating that composting method influences ion availability and organic matter interactions. Remarkably, the compost of algae (CA) deviated from the expected trend, exhibiting a higher pH than predicted based on EC values. This anomaly can be explained by the NH_4_^+^ concentration in CA (1.77 mg dm^−3^), which was significantly lower than that of the other samples (4.6 to 159 mg dm^−3^). The non-composted organic fertilizer of livestock waste (FLW) followed the same linear correlation observed for composted samples, suggesting a similar ionic balance.

Among the major elements (K, Na, Ca, Mg), Na and K were the most abundant in all DOM samples, except for compost of sewage sludge (CSS), where K and Mg were found in similar concentrations (Table S2). None of the extracts exhibited excessive Na levels that could compromise compost use as a soil amendment, considering the role of Na in inhibiting Ca and Mg absorption [17]. When comparing CDDW and CVDW, which originated from the same feedstock, their major element concentrations were remarkably similar, reinforcing the hypothesis that feedstock composition plays a major role in nutrient availability. Furthermore, the non-composted organic fertilizer (FLW) exhibited comparable values as composted samples, suggesting that composting does not significantly alter the overall chemical composition of these major elements.

- Spectroscopic Characterization

The molar absorptivity coefficient at 280 nm (*ε*_280_), a proxy for aromatic content, was calculated for each equilibrium solution (Table S3). These values were used to estimate relative aromaticity and average molar mass of the dissolved organic matter extract (DOM), based on empirical relationships [18]:

Aromaticity (%) = 0.05 *ε*_280_ + 6.74 (1)

Molar mass (g mol^−1^) = 3.99 *ε*_280_ + 490 (2)

For most DOM extracts, except for CSS, the aromaticity ranged from 20% to 27%, which is consistent with the presence of hydrophobic and recalcitrant moieties that are resistant to degradation. These results were expected, since these composts have undergone an extensive maturation process [19]. Particularly, the highest aromaticity and molecular mass values were observed in the non-composted FLW, likely due to both the nature of the feedstock and the biochemical transformations occurring during the digestive process. This suggests that animal digestion can enhance the production of aromatic moieties, resulting in a material with a higher degree of aromatic condensation than composted samples.

#### 2.1.2. Humic-like Substances

- Physico-Chemical Characterization

The extraction yields and elemental composition of HS-L are reported in Table S4. The total HS-L yield was highest in CLW (41.9 g kg^−1^ compost) and lowest in CA (22.5 g kg^−1^ compost), illustrating substantial variability across samples. The non-composted FLW exhibited the highest yield (64.5 g kg^−1^ compost), surpassing all composted samples.

The C/N, O/C, and H/C atomic ratios, commonly used to classify HS-L fractions, are plotted in a van Krevelen diagram (Figure S1a) and bar chart (Figure S1b)**.** As expected, HA-L fractions exhibited lower O/C ratios compared to FA-L, reflecting their higher carbon content and lower oxygen content. This difference is consistent with their solubility characteristics, as FA-L is soluble across a broader pH range, whereas HA-L solubility is limited to alkaline conditions (pH > 10). The H/C ratio, an indicator of aliphatic vs. aromatic content, was generally similar between FA-L and HA-L, except in the compost of urban waste (CUW), where FA-L had a higher H/C ratio, indicating a lower degree of aromaticity compared to HA-L. The C/N ratio, an indicator of nitrogen enrichment, was generally lower in HA-L than in FA-L, suggesting a higher nitrogen retention in the humic fraction [20,21,22]. However, this trend was not observed for CVA, CSS, and FLW, which may be attributed to differences in feedstock composition and composting processes. Sewage sludge (CSS) and the non-composted organic fertilizer (FLW) likely contain inorganic nitrogen forms that are less effectively incorporated into humic structures, while vermicomposting (CVA) may have influenced a differential transformation of organic matter, affecting nitrogen distribution between fractions.

- Spectroscopic Characterization

Molar absorptivity coefficients (*ε*_280_) were determined for FA-L and HA-L (Table S3), providing insights into their aromaticity and molecular mass [18]. HA-L fractions consistently exhibited higher *ε*_280_ values than FA-L, indicating greater aromatic content. The only exceptions were CVA and CA, where both fractions had similar values. A comparison between DOM and HS-L showed that DOM values were generally lower than those of HA-L, except in CSS and FLW, where DOM exhibited comparable aromaticity levels. FA-L fractions could be categorized into two groups: those with higher aromaticity than DOM (CVA, CA, CUW) and those with lower aromaticity than DOM (CVDW, CLW, CDDW, CSS, and FLW).

The molecular composition of HA-L and FA-L was further characterized by ^1^H-NMR spectroscopy. The samples were prepared with deuterated water, and the *pH* was adjusted to 12 with 40% NaOD. The spectra were divided into five regions, corresponding to different proton environments: C-alkyl and N-alkyl protons (δ 0–3.0 ppm); O-alkyl, including phenolic protons (δ 3.0–4.5 ppm); H_2_O protons (δ 4.7–5.0 ppm); alkenes and CH_2_ groups linked to aromatic rings (δ 5.0–6.0 ppm); alkene and aromatic protons (δ 6.0–9.0 ppm). To assess the relative contribution of aliphatic and aromatic structures, the ratio between the integration areas of aliphatic protons (δ 0–4.5 ppm) and aromatic protons (δ 6.0–9.0 ppm) was calculated. The results (Table S5) indicate that this ratio was generally higher in HA-L than in FA-L, except in CDDW, where both fractions had similar values, and in CA, where FA-L exhibited a higher aromatic/aliphatic proton ratio (H_aro_/H_ali_) than HA-L. Interestingly, the similarity in these ratios between HA-L and FA-L from CLW and FLW suggests a common origin, as both samples were derived from manure, despite FLW not undergoing composting.

The ATR-FTIR spectra of HS-L extracts from different composts (Figure S2) revealed characteristic absorption bands associated with their molecular structures. The broad band at 3600–3000 cm^−1^ was assigned to O–H stretching, likely from phenols, alcohols, and carboxyl groups, as well as N–H stretching from amides and amines [23,24,25]. The presence of two bands at 2925 and 2845 cm^−1^ confirmed the occurrence of asymmetric and symmetric C–H stretching in aliphatic CH_3_ and CH_2_ groups [25,26,27]. A shoulder at 1700 cm^−1^, corresponding to C=O stretching of carboxyls and ketones [25,26,27,28], was observed in all extracts. In addition, a band at 1640–1630 cm^−1^ was attributed to aromatic C=C stretching, as well as C=O stretching in amides and quinones [25,26]. The bands at 1530–1520 cm^−1^ suggested additional contributions from aromatic C=C bonds and/or N–H bending vibrations and C=N stretching (amides II) [4,25,26,27,28]. Differences among samples were also noted in the 1400–1220 cm^−1^ region, where variations in C–H deformations, aryl ether stretching, and carboxyl O–H bending were observed [4,25,26,27,28].

The most significant spectral differences between FA-L and HA-L appeared in the 3700–2700 cm^−1^ and 1800–1000 cm^−1^ regions, particularly in the relative intensities of the 1700 cm^−1^ (C=O) and 1630 cm^−1^ (C=C) bands. In all FA-L samples, except for CSS, the 1700 cm^−1^ band was either equal in intensity or more prominent than the 1630 cm^−1^ band. In contrast, the opposite trend was observed for HA-L, where the C=C band at 1630 cm^−1^ was consistently stronger, indicating greater aromatic character in these fractions.

To further quantify these differences, aromaticity indices (I_1630/2925_ and I_1630/2845_) were calculated (Table S6) [24]. Based on these values, HA-L samples were grouped into three categories: CSS had the lowest aromaticity, CVA, CLW, CUW, and CDDW exhibited intermediate values, while CA showed the highest aromaticity. In FA-L, a different trend was observed, with CVDW displaying the lowest values, CLW, CA, CVA, CDDW, and CSS falling into an intermediate range, and CUW exhibiting the highest aromaticity. Overall, HA-L from CA and FA-L from CUW are the most aromatic among all samples, highlighting compositional differences that may influence their functionality and reactivity.

- Thermal Characterization

**Figure 2:**
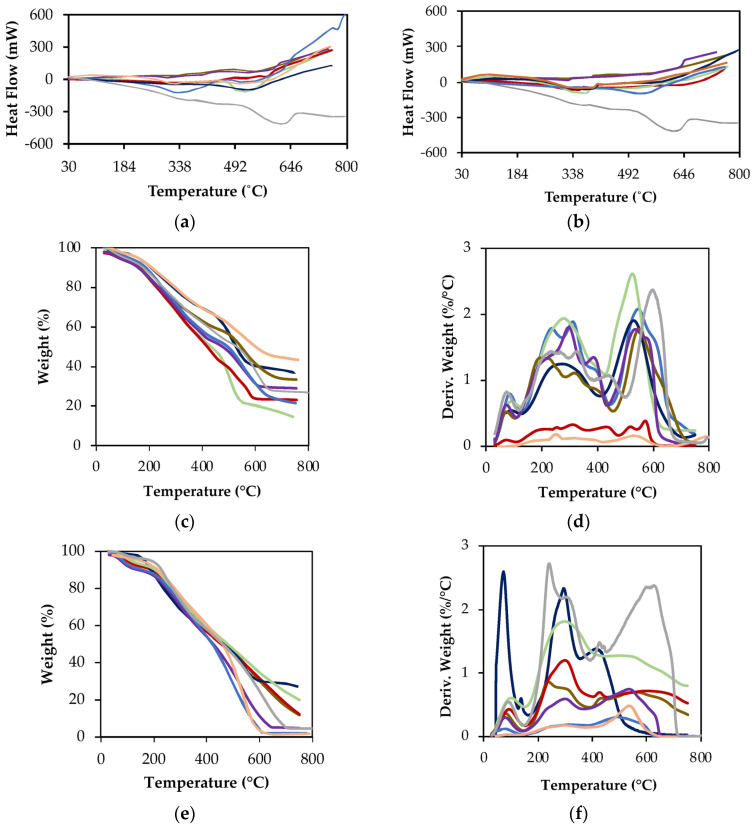
DSC curves of (**a**) FA-L and (**b**) HA-L and TGA (**c**,**e**) and DTG (**d**,**f**) curves obtained in air atmosphere for (**c**,**d**) FA-L and (**e**,**f**) HA-L extracted from the samples: CDDW (purple), CVDW (red), CVA (green), CA (orange), CUW (blue), CSS (dark blue), CLW (brown) and FLW (gray).

The DSC curves of HS-L from different samples (Figure 2) exhibit noticeable variations in shape and peak intensity; however, they share common thermal features. A minor endothermic band is observed at ~100 °C, typically attributed to dehydration. Additionally, two broad exothermic bands appear at ~320 °C and ~500 °C, corresponding to the decomposition of recalcitrant and extra-recalcitrant organic carbon, respectively.

The enthalpy values obtained from curve integration across three temperature ranges—30–177 °C (*H*_1_), 177–400 °C (*H*_2_), and 400–620 °C (*H*_3_)—are summarized in Table S7. A key observation for FA-L extracts is that the enthalpy of extra-recalcitrant decomposition (*H*_3_) is equal to or greater than that of recalcitrant substances (*H*_2_) in all samples, except for CDDW and CSS. In contrast, for HA-L, *H*_2_ is generally equal to or higher than *H*_3_, except for CUW and CSS, where the opposite trend is observed.

The TGA and DTG curves of FA-L (Figure 2c,d) and HA-L (Figure 2e,f) extracts provide further insights into the thermal stability of these materials. Under an oxidative atmosphere, three primary weight loss (WL) stages were identified: *WL*_1_ (30–177 °C), *WL*_2_ (177–400 °C), and *WL*_3_ (400–620 °C). These correspond to *H*_1_ (labile compounds), *H*_2_ (recalcitrant organic compounds), and *H*_3_ (extra-recalcitrant organic compounds), respectively.

The weight loss values at the different temperature ranges, as well as the total weight loss (*TWL*) are summarized in Table S7 for the different samples. The lowest *TWL* for FA-L was recorded in CA (59.8%), whereas CVA exhibited the highest value (84.5%). For HA-L, the lowest *TWL* was observed in CSS (66.9%), while the highest values were recorded for CUW (97.9%) and CDDW (93.9%) (Figure 2c,e). These findings suggest a direct relationship between *TWL* and feedstock composition, as CDDW and CUW—both derived predominantly from vegetable sources—exhibited similar *TWL* values.

The DTG curves (Figure 2d,f) indicate that each weight loss stage (*WL*_1_, *WL*_2_, and *WL*_3_) likely results from the overlap of multiple decomposition processes, as evidenced by broad, asymmetric peaks. This effect is particularly noticeable in the second decomposition stage (*WL*_2_), where peak profiles are highly irregular. Among FA-L extracts, CVDW and CA displayed the most distinct thermal profiles, whereas in HA-L extracts, CSS and CVA exhibited the greatest deviations from the general trend.

The weight loss values for the three thermal regions are presented in Table S7. In the first stage (*WL*_1_: 30–177 °C), all samples showed weight loss between 1.4 ± 0.4% and 8.6 ± 2.2%, indicative of moisture loss and the decomposition of volatile compounds. For *WL*_2_, FA-L and HA-L samples can be grouped into two categories: those with higher *WL*_2_ values (CVDW, CA, and CVA) and those with lower *WL*_2_ values (CDDW, CUW, and CLW). A lower *WL*_2_ suggests a smaller proportion of labile organic structures, possibly due to feedstock composition or a more advanced composting process, which would have more effectively degraded these structures. Regarding *WL*_3_ (extra-recalcitrant fraction), CA and CUW exhibited the highest values for FA-L and HA-L extracts, whereas CVDW and CSS displayed the lowest. These results suggest that CA and CUW contain a greater proportion of highly stable, extra-recalcitrant structures, while CVDW and CSS have a lower content of these thermally resistant components.

### 2.2. Correlations Between Physico-Chemical and Reactivity Parameters

Given the large number of parameters obtained from different analytical techniques and the inherent complexity of compost samples, direct comparisons are challenging. The calculated indices do not always produce a consistent ranking of samples, likely due to overlapping signals that complicate deconvolution. However, the reliability of each parameter can be assessed by examining cross-correlations between different analytical results. Significant variations in extract composition should be reflected across multiple techniques, affecting both physicochemical properties and reactivity-related characteristics.

To explore these relationships, a correlation analysis was performed, comparing physicochemical and reactivity parameters from FA-L, HA-L, and DOM extracts (Section 2.2.1). The reactivity parameters, previously reported in earlier studies, include the following:

- The abundance of acidic functional groups (*M_i_*) and protonation constants (*K_i_*), derived from acid-base titrations [29].

For DOM, *M*_1_ and *K*_1_ are related to carboxylic groups in amino acids; *M*_2_ and *K*_2_ represent those of carboxyl groups in organic acids; *M*_3_ and *K*_3_ correspond to phenolic hydroxyl groups.

For HS-L, *M*_1_ is abundance and *K*_1_ the protonation constant of carboxylic-type groups; *M*_2_ is abundance and K_2_ the protonation constant of phenolic groups.

- The extent of Cd^2+^ binding by organic matter in the extracts, assessed at two metal concentrations—low (*c_ML_*_,*L*_, 10^−8^ mol L^−1^) and high (*c_ML_*_,*H*_, 10^−6^ mol L^−1^)—to distinguish between the strongest binding sites and the overall complexation capacity, evaluated through metal titrations [9].

Additionally, correlations were examined between the physicochemical and reactivity parameters of the extracts and the characterization parameters of the original compost samples (Section 2.2.2). The compost characterization data, previously published in [30], included the following:

- Elemental composition (C, C_oxi_, N, C/N, C_oxi_/C, S, CEC, Na, K, Fe, As, Ca, Mg, Al, Cu, Mn);
- Cation exchange capacity (*CEC*), which is a measure of the compost’s ability to hold positively charged ions;
- Spectroscopic features from UV-vis (namely *ε*_280_) and ATR-FTIR (namely *I*_1630/2925_, *I*_1630/2850_ which serves as an aromaticity index, concerning the degree of aromatic condensation in humic structures;
- Thermal properties assessed using Differential Scanning Calorimetry (DSC) (*H*_1_, *H*_2_, *H*_3_) and Thermogravimetric Analysis (TGA) (*WL*_1_, *WL*_2_, *WL*_3_, *WL*_4_) [30]:
  ▪ *H*_1_ and *WL*_1_ (30–177 °C), correspond to dehydration and desorption processes, providing insight into the affinity for low molecular weight substances;
  ▪ *H*_2_ and *WL*_2_ (177–400 °C) reflect the decomposition of recalcitrant organic matter;
  ▪ *WL*_3_ (400–620 °C) + *WL*_4_ (620–800 °C) and *H*_3_ are related to the breakdown of extra-recalcitrant structures and carbonate decomposition.

#### 2.2.1. Correlation Between the Chemical Composition and Reactivity of Extracts

The chemical composition of organic matter extracts plays a crucial role in determining their reactivity, as it influences functional group availability, molecular interactions, and structural stability. The presence and distribution of acidic functional groups (carboxyl and phenolic groups) affect protonation behavior and metal-binding capacity, while aromaticity and oxidation state influence thermal stability and electron transfer processes. Therefore, strong correlations between composition and reactivity parameters are expected, as chemically distinct extracts should exhibit systematic differences in their capacity to interact with metals, buffer pH, and undergo degradation.

- General Trends

**Figure 3:**
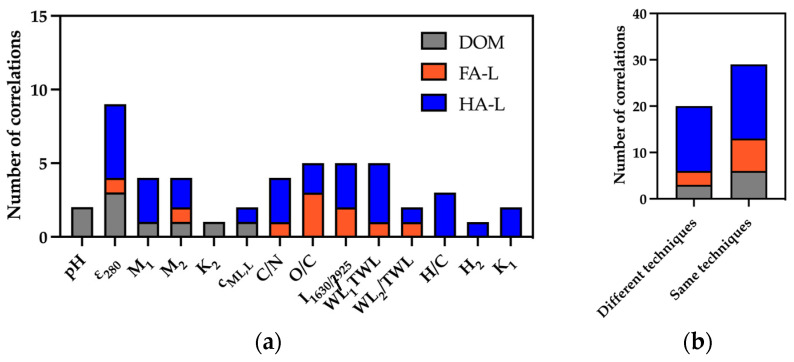
(**a**) Number of correlations found (|*r*| > 0.7) for each characterization parameter within data from FA-L, HA-L, and DOM. (**b**) Number of total correlations found for FA-L, HA-L and DOM. The identification of the parameters is provided in Table S13 in the Supplementary Information (SI).

The number of significant correlations found for each extract is summarized in Figure 3a. Among the three extracts, HA-L exhibited the highest number of correlations (30), while DOM had the lowest. Only correlations with |*r*| > 0.7 were considered in this analysis. The distinction between intra-technique and inter-technique correlations is presented in Figure 3b, highlighting the most interconnected parameters: *ε*_280_ (HA-L: 5 correlations; DOM: 3 correlations), *WL*_1_/*TWL* (HA-L: 4), *I*_1630/2925_ (HA-L: 3), C/N (HA-L: 3), and O/C (FA-L: 3).

- FA-L Correlations

**Figure 4:**
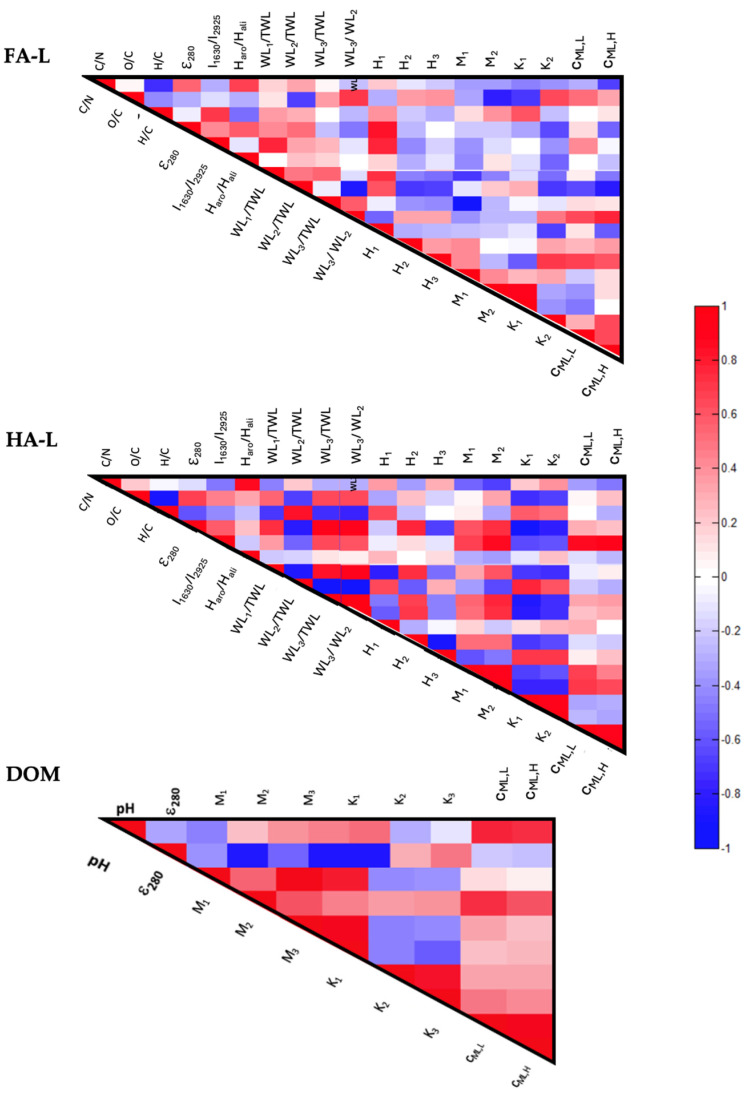
Triangular heat map showing the pairwise Pearson correlation coefficients (*r*) of the parameters from FA-L, HA-L, and DOM. The identification of the parameters is provided in Table S13 in the Supplementary Information (SI).

The correlation analysis for FA-L extracts (Figure 4) included 18 parameters from both physicochemical and reactivity characterizations. Among the ten significant correlations (|*r*| > 0.7), seven involved parameters from different techniques. These correlations can be grouped as follows:

(a) Aromaticity and Interaction with Low Molecular Weight Compounds: *ε*_280_ and *I*_1630/2925_ positively correlate with *H*_1_ and *WL*_1_/*TWL*, suggesting that more aromatic FA-L fractions exhibit stronger interactions with low molecular weight substances. While *WL*_1_ (30–177 °C) is primarily associated with moisture loss and volatile compound desorption, the observed correlation with aromaticity parameters may indicate that highly aromatic FA-L fractions provide a greater number of weakly bound interaction sites, leading to an increased release of volatile organic compounds during the first thermal stage. This suggests that, although *WL*_1_ does not directly quantify adsorption capacity, it may reflect the degree to which small molecules interact with and are retained by FA-L structures before being thermally desorbed.
(b) Oxygen Content and Affinity Distributions: O/C correlates positively with *K*_1_, indicating that samples with a higher oxygen content tend to have carboxyl groups with stronger protonation constants (higher *K*_1_ values). This suggests that oxygen-rich FA-L fractions contain a greater proportion of carboxyl functional groups that exhibit a stronger affinity for protons (H^+^), likely due to increased acidity or structural differences in the organic matrix. The enhanced presence of oxygen-containing functional groups may contribute to greater reactivity in acid-base interactions, influencing the overall buffering capacity and metal-binding properties of FA-L.
(c) Cd^2+^ Binding and Mass Loss: *c_ML_*_,*H*_ correlates with *WL*_2_/*TWL*, suggesting that the organic structures responsible for Cd^2+^ binding are also involved in the thermal degradation of biodegradable aromatic moieties. Specifically, this implies that carboxyl- and phenol-rich functional groups, which strongly complex Cd^2+^ at high concentrations (*c_ML_*_,*H*_), may be associated with organic matter components that decompose in the 177–400 °C range (*WL*_2_). These structures likely include aromatic moieties linked to carbohydrates or aliphatic chains, which are more thermally labile than fully condensed aromatic domains. The correlation indicates that Cd^2+^ binding sites may be partially lost during thermal degradation, reflecting the reactivity of these functional groups in organic matter transformation processes.
(d) Oxygen Content and Aromatic Stability: O/C negatively correlates with *WL*_2_/*TWL* and *M*_2_, suggesting that higher oxygen content is associated with a decrease in biodegradable aromatic structures and phenolic groups. This may indicate oxidation-driven conversion of aromatic moieties into more recalcitrant structures, possibly through phenolic-to-carboxyl transformations or mineralization to carbonates or CO_2_.

- HA-L Correlations

For HA-L (Figure 4), the analysis also included 18 parameters, revealing thirty significant correlations (|*r*| > 0.7), of which sixteen were inter-technique correlations. These correlations were grouped into the following key relationships:

(a) Aromaticity and Nitrogen Content: a positive correlation between C/N and *H_aro_*/*H_ali_* suggests that higher aromaticity (as assessed by ^1^H-NMR) is associated with lower nitrogen content in HA-L. This implies that as humic structures become more condensed and aromatic, nitrogen incorporation decreases, likely because nitrogen-rich compounds (e.g., proteins, peptides) degrade more readily during humification and are preferentially retained in less aromatic fractions.
(b) Condensation and Degradability: The positive correlation between H/C with *WL*_2_/*TWL* indicates that higher H/C ratios (signifying less condensed structures) are associated with a greater abundance of biodegradable aromatic moieties. This suggests that less condensed HA-L fractions retain more aliphatic and labile aromatic components, which decompose in the 177–400 °C range (*WL*_2_), whereas highly condensed structures are more resistant to thermal degradation.
(c) Aromaticity, Thermal Stability, and Interaction with Small Molecules: The positive correlation between *ε*_280_ and *WL*_1_/*TWL*, *WL*_3_/*TWL*, and *H*_2_ suggests that highly aromatic HA-L fractions are more thermally stable and exhibit enhanced interactions with small organic molecules. The correlation with *WL*_1_ (30–177 °C) indicates that aromatic HA-L structures may retain weakly bound volatile compounds, which are released at lower temperatures. Similarly, the correlation with *WL*_3_ (400–620 °C) implies that more condensed aromatic structures contribute to the retention of thermally stable components, which decompose only at higher temperatures. The association with *H*_2_ (177–400 °C) further suggests that aromatic HA-L fractions participate in exothermic reactions within this temperature range, possibly due to the controlled degradation of structurally stable organic moieties.
(d) Aromaticity, Phenolic Groups, and Cd^2+^ Binding: *I*_1630/2925_ and *ε*_280_ correlate positively with *M*_2_, *c_ML_*_,*L*_, and *c_ML_*_,*H*_, indicating that higher aromaticity is linked to a greater abundance of phenolic functional groups and stronger metal-binding capacity. Since phenolic groups are known to contribute significantly to metal complexation, this correlation suggests that aromaticity enhances the density of reactive sites for metal interaction, increasing the binding affinity for Cd^2+^.
(e) Cd^2+^ Binding and Carboxyl Group Reactivity: The positive correlation between *K*_1_ and *c_ML_*_,*L*_ demonstrates that Cd^2+^ affinity is closely related to carboxyl group reactivity. This suggests that carboxyl sites with stronger acid dissociation constants (higher *K*_1_ values) contribute more significantly to Cd^2+^ complexation, particularly at low metal concentrations.

Negative correlations further reinforced these trends:

(f) Aromatic Condensation and Molecular Interaction Capacity: The negative correlation between H/C and *WL*_3_/*TWL* suggests that as HA-L becomes more condensed and aromatic (lower H/C), it contains a higher proportion of recalcitrant structures that degrade at higher temperatures (*WL*_3_). Additionally, the negative correlation between H/C and *WL*_1_/*TWL* indicates that these condensed domains may also retain small organic molecules, contributing to mass loss at lower temperatures (*WL*_1_). This suggests that strong π–π interactions and hydrophobic forces within highly aromatic HA-L fractions promote both structural stability and the retention of thermolabile compounds.
(g) Aromaticity and Oxidation: The negative correlation between O/C and *WL*_2_/*TWL* suggests that higher oxidation levels reduce the abundance of easily biodegradable aromatic moieties. This implies that as HA-L undergoes oxidation, it becomes more chemically stable and less prone to thermal degradation in the 177–400 °C range (*WL*_2_), meaning that fewer labile aromatic structures remain in the extract.
(h) Aromaticity and Recalcitrance: The negative correlation between *ε*_280_ and *WL*_2_/*TWL* indicates that more aromatic HA-L fractions contain fewer recalcitrant structures. This suggests that samples with higher aromaticity have undergone greater structural transformation, leading to a reduction in less-condensed, thermally labile components that degrade in the 177–400 °C range.
(i) Nitrogen Content and Acidic Functional Group Reactivity: The negative correlation between C/N and *K*_1_ and *K*_2_ suggests that higher nitrogen content enhances the reactivity of both carboxylic (*K*_1_) and phenolic (*K*_2_) groups. This implies that as nitrogen-rich structures are incorporated into HA-L, they contribute to more reactive acidic functional groups, increasing their ability to participate in protonation and metal-binding interactions.

- DOM Correlations

For DOM (Figure 4), the analysis included 10 parameters, revealing nine significant correlations (|*r*| > 0.7), six of which involved different techniques. The main trends observed were:

(a) Aromaticity and Carboxyl Reactivity: The positive correlation between *ε*_280_ and *K*_1_ suggests that more aromatic DOM fractions contain carboxyl groups with stronger acidity (lower protonation constants), making them more reactive. This implies that aromaticity is linked to the presence of highly dissociable carboxyl functional groups, enhancing proton exchange capacity in DOM.
(b) pH and Cd^2+^ Binding: The positive correlation between pH and *c_ML_*_,*L*_, *c_ML_*_,*H*_, indicates that higher pH enhances the ability of DOM to bind Cd^2+^, likely due to the increased deprotonation of acidic functional groups. As pH rises, more carboxyl and phenolic sites lose protons, increasing their negative charge and strengthening metal complexation interactions.
(c) Carboxylic Group Distribution and Cd^2+^ Binding: The positive correlation between *M*_2_ (carboxyl groups in organic acids) and *c_ML_*_,*L*_ suggests that organic acid-derived carboxyl groups are key contributors to Cd^2+^ binding in DOM, particularly at low metal concentrations. This implies that these functional groups provide effective coordination sites for Cd^2+^ complexation, enhancing the metal-binding reactivity of DOM.
(d) Aromaticity and Functional Groups: The negative correlation between *ε*_280_ correlates and *M*_1_, *M*_3_ suggests that highly aromatic DOM fractions contain fewer amino acid-associated carboxyl groups (*M*_1_) and phenolic structures (*M*_3_). This implies that as DOM becomes more structurally condensed and aromatic, it may undergo selective depletion of phenolic functionalities, potentially altering its overall reactivity.

#### 2.2.2. Correlation Between the Composition of the Extracts and the Original Compost

The composition of the original compost significantly influences the characteristics of its organic matter extracts, as it dictates the chemical nature, structural stability, and functional group distribution of the dissolved and humic-like fractions. The relative abundance of aromatic, aliphatic, and oxygen-containing moieties in the bulk compost affects the formation, solubility, and reactivity of extracted organic matter, shaping its acid-base behavior, metal-binding properties, and thermal stability.

To further explore these relationships, three correlation analyses were performed, examining the composition of HA-L, FA-L, and equilibrium solutions (containing DOM) in relation to compost characterization parameters. These analyses aim to identify predictive links between compost composition and extract reactivity, offering insights into how bulk compost properties translate into the physicochemical and functional traits of the extracted fractions.

- DOM vs. Compost

**Figure 5:**
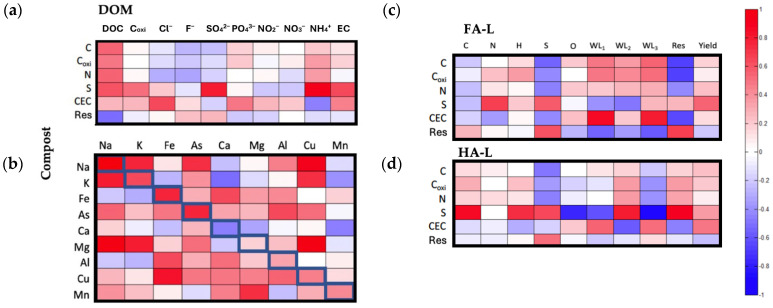
Heat map showing the pairwise Pearson correlation coefficients (*r*) of (**a**) the characterization parameters of DOM vs. relatable parameters of compost, (**b**) metal content in DOM vs. metal content of compost, (**c**) the characterization parameters of FA-L vs. relatable parameters of compost and (**d**) the characterization parameters of HA-L vs. relatable parameters of compost. The cells highlighted in diagonal in (**b**) correspond to the correlations between the same metal in the two samples. The identification of the parameters is provided in a Table S13 in the Supplementary Information (SI).

The first correlation analysis (Figure 5a) examined the relationship between 10 parameters from DOM and 6 compost parameters (bolded in the figure). Two significant positive correlations (|*r*| > 0.7) were identified:

- Sulfur (S) in compost and SO_4_^2−^ and NH_4_^+^ in equilibrium solutions: This correlation suggests a strong association between compost sulfur content and sulfate and ammonium concentrations in solution. While the correlation between S and SO_4_^2−^ is expected, the link between S and NH_4_^+^ is less straightforward and requires further investigation.

The second correlation analysis (Figure 5b) focused on metal mobility, assessing how metal concentrations in DOM relate to their total content in compost. Significant positive correlations (*r* > 0.7) were observed for Na, K, Fe, and As, suggesting that their concentrations in solution are strongly influenced by their abundance in compost. This trend implies that these elements are either present in readily extractable forms or subject to dynamic equilibrium between bound and free states.

For the remaining metals, binding interactions with HS-L appear to be the dominant factor, resulting in lower correlation coefficients. Additionally, certain metals in equilibrium solutions (e.g., Cu or K) correlated with Na, K, and Mg in compost. These associations likely reflect compositional similarities in the raw materials used for composting, influencing the overall elemental distribution in both the solid and dissolved phases.

- HS-L vs. Compost

The third correlation analysis evaluated the relationship between FA-L and HA-L composition and compost properties (Figure 5c,d). Two significant correlations were identified for FA-L, while six correlations were found for HA-L (|*r*| > 0.7).

For FA-L (Figure 5c), two positive correlations were observed:

(a) Cation Exchange Capacity and Thermal Stability of FA-L: The positive correlation between *CEC* and *WL*_3_ suggests that composts with higher cation exchange capacity (*CEC*) contain FA-L fractions enriched in extra-recalcitrant moieties, which may contribute to long-term soil fertility and metal-binding properties. Additionally, the correlation between *CEC* and *WL*_1_ indicates that composts with higher *CEC* tend to produce FA-L fractions with a greater capacity to retain and interact with low molecular weight substances, possibly due to the presence of reactive acidic functional groups.

For HA-L (Figure 5d), four positive correlations were identified:

(a) Sulfur (S) in compost and HA-L Composition and Thermal Stability: The positive correlation between compost S and HA-L carbon (C), hydrogen (H), *WL*_2_, and residue (*Res*) in TGA suggests that sulfur-rich composts yield HA-L fractions with greater organic carbon and hydrogen content, a higher proportion of recalcitrant structures, and increased thermal stability. This could indicate that sulfur incorporation during composting enhances the structural integrity and stability of humic-like substances.

Additionally, two negative correlations were observed:

(b) Sulfur (S) in compost and Oxygen Content and Recalcitrance in HA-L: The negative correlation between S in compost and O in HA-L suggests that higher sulfur content is associated with a reduced presence of oxygen-containing functional groups in HA-L. Simultaneously, the negative correlation between S and *WL*_3_ indicates that sulfur-rich composts yield HA-L fractions with fewer extra-recalcitrant structures. This suggests a structural modification of humic-like substances, where sulfur incorporation may alter oxidation pathways, potentially influencing the composition and long-term stability of HA-L.

These findings suggest that composts with higher sulfur content yield HA-L fractions with lower oxygen content and fewer extra-recalcitrant structures. This inverse relationship implies that sulfur incorporation during composting may alter the oxidation state of organic matter, reducing oxygen-containing functional groups and potentially affecting the stability and reactivity of humic-like substances.

#### 2.2.3. Correlation Between Structural and Reactivity Features of Extracts and Compost Molecular Structure

**Figure 6:**
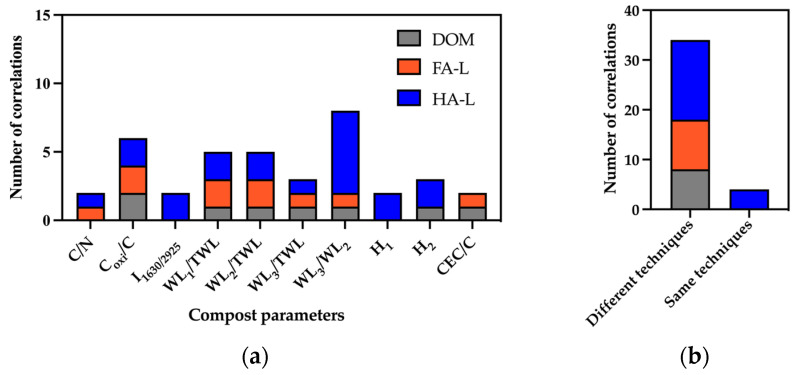
(**a**) Number of correlations between characterization parameter of FA-L, HA-L, and DOM with the characterization parameters of compost and (**b**) number of total correlations found for FA-L, HA-L and DOM. The identification of the parameters is provided in Table S13 in the Supplementary Information (SI).

In this final section, the composition of solid compost was used to perform three correlation analyses, relating compost properties to the reactivity of FA-L, HA-L, and DOM extracts. Figure 6 summarizes the number of significant correlations found for each compost parameter across the different extracts. Among the extracts, HA-L exhibited the highest number of correlations (20), while DOM showed the lowest connectivity with compost parameters.

- DOM vs. Compost

**Figure 7:**
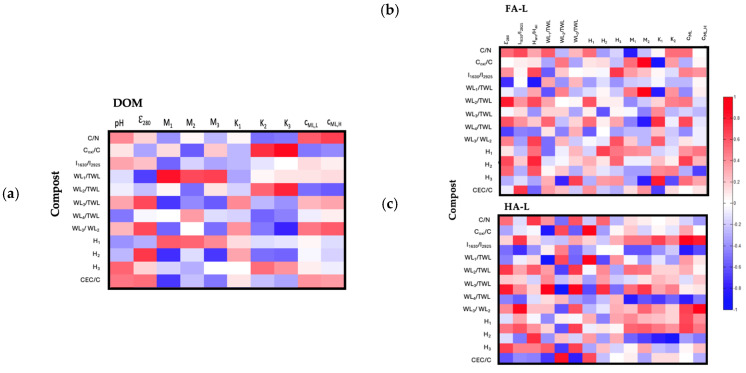
Heat map showing the pairwise Pearson correlation coefficients (*r*) of (**a**) the parameters from DOM vs. compost, (**b**) the parameters from FA-L vs. compost and (**c**) the parameters from HA-L vs. compost. The identification of the parameters is provided in Table S13 in the Supplementary Information (SI).

The correlation analysis between DOM and compost included 12 compost parameters (illustrated in Figure 7) and 10 parameters from DOM. Four positive correlations were identified, categorized into two groups:

(a) Carboxyl and Phenolic Group Reactivity in DOM and Readily Oxidizable Carbon: The positive correlation between *K*_2_ and *K*_3_ vs. *C_oxi_*/*C* suggests that highly reactive carboxyl and phenolic groups in DOM are associated with composts that contain a higher proportion of readily oxidizable carbon relative to total carbon. This indicates that oxidizable carbon plays a key role in enhancing the functional reactivity of DOM.
(b) Amino Acid-Associated Carboxyl Groups and Phenolic Reactivity in DOM and Compost Thermal Stability: The positive correlation between *M*_1_ and (*WL*_1_/*TWL*) suggests that DOM fractions with a higher abundance of amino acid–associated carboxyl groups (*M*_1_) originate from composts with an increased ability to adsorb low molecular weight substances. Similarly, the correlation between *K*_3_ and *WL*_2_/*TWL* indicates that highly reactive phenolic groups (*K*_3_) are more prevalent in DOM extracted from composts with a greater proportion of recalcitrant structures.

Additionally, four negative correlations were found:

(c) Amino Acid-Associated Carboxyl Groups and Compost Maturity Indicators: The negative correlations between *M*_1_ and *WL*_3_/*TWL*, *H*_2_, and *CEC*/*C* suggest that DOM fractions with a higher abundance of amino acid–associated carboxyl groups (*M*_1_) originate from composts with lower maturation indices (i.e., lower *CEC*/C, *WL*_3_/*TWL* and *H*_2_). This indicates that as compost matures, the contribution of amino acid–associated functional groups in DOM decreases.
(d) Phenolic Group Reactivity and Compost Maturation Degree: The negative correlation between *K*_3_ and *WL*_3_/*WL*_2_ suggests that DOM fractions enriched in highly reactive phenolic groups (*K*_3_) are more prevalent in composts with lower maturation degrees. This implies that as compost matures, phenolic structures in DOM undergo transformations, reducing their reactivity.

- HS-L vs. Compost

The correlation analysis between HS-L and compost (Figure 7b,c) included 10 parameters from FA-L and HA-L extracts and 12 compost parameters. A total of ten significant correlations were identified for FA-L and 16 for HA-L (|*r*| > 0.7), all involving parameters from different analytical techniques.

With respect to FA-L (Figure 7b) four positive correlations were identified:

(a) Phenolic Group Abundance and Oxidizable Carbon and Thermal Stability of Compost: The positive correlation between *M*_2_ and *C_oxi_*/C, *WL*_2_/*TWL* suggests that FA-L fractions rich in phenolic groups originate from composts with higher readily oxidizable carbon content, which decomposes between 177 and 400 °C.
(b) Aromaticity and Recalcitrant Structures of Compost: The positive correlation between ε_280_ and *WL*_3_/*TWL* indicates that more aromatic FA-L fractions (evaluated by ε_280_) are derived from composts with a greater abundance of extra-recalcitrant structures.
(c) Aromaticity and Cation Exchange Capacity of Compost: The positive correlation between *H_aro_*/*H_ali_* and *CEC*/C demonstrates that the aromaticity of FA-L (assessed by ^1^H-NMR) is linked to composts with a greater ability to exchange cations per unit of carbon.

Simultaneously, six negative correlations were observed for FA-L:

(d) Carboxyl Group Content and Compost C/N Ratio of Compost: The negative correlation between *M*_1_ and C/N suggests that FA-L fractions with a higher content of carboxyl groups (*M*_1_) originate from composts with a greater nitrogen content relative to carbon.
(e) Carboxyl Group Reactivity and Oxidizable Carbon of Compost: The negative correlation between *K*_1_ and *C_oxi_*/C, *WL*_2_/*TWL* suggests that more reactive carboxyl groups in FA-L are found in composts with lower readily oxidizable carbon content.
(f) Aromaticity and Compost Adsorption of Low Molecular Weight substances: The negative correlations between *ε*_280_ and *WL*_1_/*TWL*, as well as *H_aro_*/*H_ali_* and *WL*_1_/*TWL*, indicate that FA-L fractions with higher aromaticity contain fewer adsorbed low molecular weight substances.
(g) Phenolic Group Abundance and Compost Maturity: The negative correlation between *M*_2_ and *WL*_3_/*WL*_2_ suggests that phenolic-rich FA-L fractions are more prevalent in composts with lower maturation degrees.

For HA-L (Figure 7c), eleven positive correlations were observed:

(a) Aromaticity and Nitrogen Content in Compost: The positive correlation between *H_aro_*/*H_ali_* and C/N indicates that aromaticity in HA-L (evaluated by ^1^H-NMR) is higher in composts with lower nitrogen content relative to carbon.
(b) Low Molecular Weight Adsorption in HA-L and Readily Oxidizable Carbon in Compost: The positive correlations between *H*_1_ and *C_oxi_*/C, *WL*_2_/*TWL* suggest that HA-L fractions with a higher adsorption capacity for low molecular weight substances originate from composts with a greater content of readily oxidizable carbon.
(c) Aromaticity and Compost Maturity: The positive correlations between *ε*_280_ and *WL*_3_/*TWL*, *WL*_3_/*WL*_2_, and *I*_1630/2925_ and *H*_2_ indicate that aromaticity in HA-L (evaluated by UV-vis) is greater in composts with a higher proportion of extra-recalcitrant structures and a greater degree of maturation.
(d) Thermal Stability of Biodegradable Aromatic Structures and Phenolic Group Abundance, and Compost Maturation: The positive correlations between *H*_2_ and *WL*_3_/*WL*_2_, and *M*_2_ and *WL*_3_/*WL*_2_ suggest that HA-L fractions with higher thermal stability of biodegradable aromatic structures (*H*_2_) and a greater abundance of phenolic groups (*M*_2_) are associated with composts that have undergone a higher degree of maturation.
(e) Cd^2+^ Binding and Aromaticity: The positive correlations between *c_ML_*_,*H*_ and *H*_2_, *c_ML_*_,*L*_ and *I*_1630/2925_, and *c_ML_*_,*H*_ and *I*_1630/2925_ indicate that HA-L fractions with a stronger capacity to bind Cd^2+^ are linked to composts with a higher proportion of aromatic structures (*I*_1630/2925_) and a greater thermal stability of recalcitrant materials (*H*_2_).

Also, five negative correlations were observed for HA-L:

(f) Low-Temperature Mass Loss and Readily Oxidizable Carbon in Compost: The negative correlation between *WL*_1_/*TWL* and *C_oxi_*/C suggests that HA-L fractions from composts with lower readily oxidizable carbon (*C_oxi_*/C,) exhibit reduced low-temperature weight loss (*WL*_1_/*TWL*). This indicates that as composts mature and lose their labile, easily oxidized carbon, the resulting HA-L fractions contain fewer volatile, weakly bound organic compounds that desorb at lower temperatures.
(g) Aromaticity and Low Molecular Weight Adsorption: The negative correlation between *I*_1630/2925_ and *WL*_1_/*TWL* reinforces that aromaticity in HA-L is higher in composts with a lower adsorption capacity for low molecular weight substances.
(h) Carboxyl Group Content, Cd^2+^ Binding, and Adsorption Capacity: The negative correlations between *M*_1_ and *H*_1_, *c_ML_*_,*L*_ and *H*_1_, and *c_ML_*_,*L*_ and *WL*_1_/*TWL* indicate that HA-L fractions with a greater abundance of carboxyl groups and stronger Cd^2+^ binding sites originate from composts with a lower capacity to adsorb low molecular weight substances.

The correlation analyses in this study reveal new opportunities for simplifying compost characterization by identifying key compost parameters that predict the composition and reactivity of HS-L. These findings are particularly relevant because HS-L (FA-L and HA-L) are the main bioactive components of compost, responsible for nutrient retention, soil conditioning, and metal-binding capacity. However, their extraction and purification are time-consuming, making routine assessment difficult.

A central finding of this study is that certain bulk compost properties provide reliable indicators of HS-L characteristics, suggesting that direct compost analysis could be a substitute for labor-intensive HS-L extractions. Specifically, the following are true:

(a) Compost oxidation state (*C_oxi_*/*C*) correlates with HS-L aromaticity and stability, indicating that compost maturity predicts humic quality.
(b) Thermal stability parameters (*WL*_3_/*WL*_2_) reflect the recalcitrance of organic matter, which influences the long-term persistence of HA-L and FA-L in soils.
(c) Cation exchange capacity (CEC) is linked to FA-L composition, meaning that composts with higher CEC yield FA-L fractions with stronger adsorption potential for nutrients and organics.
(d) Sulfur content in compost correlates with HA-L elemental composition, implying that basic compost elemental analysis could reveal humic structural properties.
(e) Aromaticity indices (*ε*_280_, *I*_1630/2925_, *H_aro_*/*H_ali_*) strongly correlate with HS-L reactivity and metal-binding potential, reinforcing their importance for soil fertility applications.

Beyond its analytical robustness, this methodology offers a cost-effective and scalable approach to compost quality assessment. Its applicability in both research and industry settings highlights its potential to improve waste management strategies and ensure the production of high-quality compost. In the long term, this approach could support the development of standardized assessment frameworks, fostering more sustainable agricultural and environmental practices. These findings suggest that adopting a correlation-based approach could bridge the gap between scientific compost characterization and practical implementation in large-scale composting operations.

Additionally, these insights align with our previous study [9], which developed a Compost Quality Index (CQI) based on HS-L reactivity and its effect on plant growth (lettuce bioassay). The CQI showed that composts with highly reactive HS-L fractions exhibited stronger bioactivity, directly influencing plant development. The present study builds on these findings by identifying the physicochemical and thermal parameters that best predict HS-L bioactivity, reinforcing the validity of using oxidation state, aromaticity indices, and thermal decomposition as practical indicators of compost quality.

By reducing dependence on HS-L extraction and minimizing redundant analyses, this study proposes a rationalized methodology for compost evaluation, ensuring that compost quality can be assessed more efficiently without compromising scientific rigor.

## 3. Materials and Methods

### 3.1. Compost Samples and Extraction Procedures

The compost samples analyzed in this study were produced from various raw materials and composting methods, including industrial composting, domestic composting, and vermicomposting. The feedstock was predominantly of plant origin in the vermicompost of domestic waste (CVDW), in the domestic compost of domestic waste (CDDW) and in the compost of urban waste (CUW). Manure was incorporated in the vermicompost of algae (CVA), in the compost of livestock waste (CLW) and in the compost of sewage sludge (CSS) and also in the sewage sludge in CSS and algae in CVA and in the compost of algae (CA). Detailed information on raw material composting procedure is compiled in Table S1. For comparative purposes, a non-composted organic fertilizer (FLW), primarily composed of chicken manure, was included in the study.

#### Extraction of Organic Matter (OM) Fractions

Three fractions were extracted from each compost sample: Dissolved Organic Matter (DOM)—representing the water-soluble fraction of compost and Fulvic Acid-Like (FA-L) and Humic Acid-Like (HA-L) Fractions—extracted using alkaline conditions.

- DOM was extracted following the protocol in López et al. (2021) [29]. Briefly, 2.50 g of compost was mixed with 50 mL of ultrapure water at natural pH in an open system and equilibrated for 5 days. The supernatant was collected by centrifugation (6000 rpm, 20 min) and stored for analysis.
- HS-L (FA-L and HA-L) were extracted using the International Humic Substances Society (IHSS) protocol, as detailed in Silva et al. (2022) [30]. Compost samples were mixed with 0.1 M NaOH under a N_2_ atmosphere at a 10:1 (v/w) ratio. The extract was acidified to pH 1 with 6 M HCl, precipitating HA-L while leaving FA-L in solution. The HA-L purification was achieved through the washing of the precipitate with HCl/HF solution to remove mineral impurities and was then dialyzed. The supernatant was purified using XAD-8 resin, followed by cation exchange resin treatment to obtain the FA-L fraction [31].

### 3.2. DOM and HS-L Characterization

The elemental and chemical composition of DOM, FA-L, and HA-L fractions were characterized using standardized methods with the complete dataset previously reported in López et al. (2021) [29] and Silva et al. (2022) [5,30]. The relevant data can be found in Tables S2 and S4.

Spectroscopic techniques were employed to evaluate the molecular structure and functional group composition of DOM, FA-L, and HA-L. Spectroscopic data are compiled in the Supporting Information (Tables S3, S5 and S6).

- Molar absorptivity at 280 nm (*ε*_280_) [18] of DOM, FA-L, and HA-L fractions was determined in accordance with the methodology outlined in López et al. (2021) [29]. Data from these analyses were partially published in Silva et al. (2022) and López et al. (2021) [5,29].
- ATR-FTIR spectra of FA-L and HA-L (Figure S2) were acquired using a Jasco FT/IR-4100 Spectrometer to identify aromatic, carboxyl, and phenolic groups, following the methodology described in Silva et al. (2022) [5]. These spectra were used to determine the relative intensity ratios of key functional groups (e.g., *I*_1630/2925_, *I*_1630/2845_) assessing the relative amounts of carboxyl, phenolic, and aromatic groups.
- ^1^H-NMR Spectroscopy was performed using a 400 MHz Bruker Avance II NMR spectrometer for the structural characterization of FA-L and HA-L. Chemical shifts (δ) are given in parts per million (ppm), downfield from tetramethylsilane (TMS), and coupling constants (J) in hertz (Hz). The HS-L solutions were obtained by dissolving 5 mg of the sample in 800 µL of deuterated water (Merck) and the pH was adjusted to 12 with 40% NaOD (Sigma-Aldrich), following Silva et al. (2022) [5]. This analysis was used to define aromatic-to-aliphatic proton ratio (*H_aro_*/*H_ali_*), assessing structural composition of HS-L fractions.

Thermal analysis of FA-L and HA-L was conducted to evaluate exothermic transitions related to organic matter stability using Differential Scanning Calorimetry (DSC) [32] and thermal stability and decomposition patterns were further evaluated through Thermogravimetric Analysis (TGA/DTG) [33], following Silva et al. (2022) [5]. Relevant data are provided in Figure 5 and Table S7.

Cd^2+^ complexation studies (using AGNES method) were used to determine the metal-binding capacity of HS-L, as previously applied in Silva et al. (2022) [5]. Acid-base titrations were conducted for DOM, FA-L, and HA-L, evaluating protonation/deprotonation behavior using automated titration equipment, following López et al. (2021) [29]. Data from these analyses are available in López et al. (2021) and Silva et al. (2022, 2023) [5,9,29] and are summarized in the Supporting Information (Tables S8–S10).

### 3.3. Compost Characterization

The elemental and chemical composition of compost samples were characterized using standardized methods with the complete dataset previously reported in López et al. (2021) [29] and Silva et al. (2022) [5,30]. The relevant data can be found in Table S11.

Thermal analysis of compost was conducted to evaluate exothermic transitions related to organic matter stability using Differential Scanning Calorimetry (DSC) [32] and thermal stability and decomposition patterns were further evaluated through Thermogravimetric Analysis (TGA/DTG) [33], following Silva et al. (2022) [5]. Data from these analyses were partially published in Silva et al. (2022) [5,30]. Relevant data are provided in Table S12.

### 3.4. Statistical and Correlation Analyses

Pearson correlation analysis was conducted using SPSS Statistics 25, considering statistically significant correlations (*p* < 0.05). The heatmap visualization was generated using MATLAB (R2007b) to explore relationships between physicochemical properties and compost reactivity [34]. This study builds upon previous work from our group, particularly the development of a Compost Quality Index (CQI) in Silva et al. (2023) [9]. While previous research established HS-L reactivity as a bioactivity indicator, this study explores whether bulk compost properties can predict HS-L characteristics, potentially reducing the need for complex extractions.

A diverse set of parameters was employed to analyze the correlation between compost samples and the three extracted fractions, Dissolved Organic Matter (DOM), Fulvic Acid-Like (FA-L) and Humic Acid-Like (HA-L). Various characterization techniques were utilized, including physicochemical, spectroscopy, and thermal analysis, as well as the assessment of reactivity and acid-base properties.

## 4. Conclusions

This study provides a comprehensive correlation analysis linking the physicochemical properties, structural composition, and reactivity indicators of compost extracts (FA-L, HA-L, and DOM) to the composition and maturity of the bulk compost itself. By integrating multiple analytical techniques, we identified key parameters that enable a more efficient assessment of compost quality, stability, and bioactivity.

A major outcome of this study is the demonstration that direct compost characterization can serve as a reliable indicator of HS-L quality, potentially eliminating the need for labor-intensive humic substance extractions. This is particularly important in agricultural applications, where HS-L plays a critical role in soil fertility and microbial activity. The results suggest that compost oxidation state, cation exchange capacity, and thermal stability serve as strong predictors of HS-L composition, supporting their use as simplified indicators for compost maturity and agronomic potential. Additionally, the study confirms that certain physicochemical parameters are strongly interdependent, allowing for a reduction in the number of necessary analyses. The strong correlations between compost composition, HS-L properties, and DOM characteristics mean that a reduced set of targeted indicators could provide equivalent information to more extensive characterization protocols, making compost quality assessment more accessible and cost-effective. These findings also reinforce our previous study [9], which introduced the Compost Quality Index (CQI) as a tool to predict compost bioactivity. The current work provides the chemical and structural basis for the CQI, demonstrating that key compost properties can reliably predict HS-L bioactivity without requiring extensive extractions. This strengthens the case for integrating simpler physicochemical indicators into routine compost evaluation protocols.

Overall, this study paves the way for a more practical, science-based approach to compost characterization, ensuring that composted materials can be effectively assessed for sustainable agricultural and environmental applications.

## Data Availability

No new data were created or analyzed in this study. Data sharing is not applicable to this article.

## Supplementary Materials

The following supporting information can be downloaded at: https://www.mdpi.com/article/10.3390/molecules30071511/s1, Figure S1. (a) Van Krevelen diagram (O/C vs. H/C atomic ratios) and (b) C/N atomic ratio of (o) HA-L and (●) FA-L extracts of the samples: CDDW (purple), CVDW (red), CVA (green), CA (orange), CUW (blue), CSS (dark blue), CLW (brown) and FLW (gray); Figure S2. ATR-FTIR spectra of (a) FA-L and of (b) HA-L obtained from the samples: CDDW (purple), CVDW (red), CVA (green), CA (orange), CUW (blue), CSS (dark blue), CLW (brown) and FLW (gray). Table S1. Identification of the compost and fertilizer samples characterized in the present work; Table S2. Characterization results of equilibrium solutions from the compost and non-composted organic fertilizer (highlighted).; Table S3. UV-vis parameters (*ε*_280_, aromaticity and molar mass) of DOM, HA-L and FA-L from the compost and non-composted organic fertilizer (highlighted). Table S4. Physicochemical characterization parameters (C, N, H, S and O) and yield (Y) of the extractions of HA-L and FA-L from the compost and non-composted organic fertilizer (highlighted). The values have been rectified on the basis of ash content.; Table S5. ^1^H-NMR parameters (Aliphatic protons, Aromatic protons and *H_aro_*/*H_ali_*) of FA-L and HA-L from the compost and non-composted organic fertilizer (highlighted).; Table S6. ATR-FTIR parameters (*I*_1630/2845_ and *I*_1630/2925_**)** of HA-L and FA-L from the compost and non-composted organic fertilizer (highlighted).; Table S7. DSC and TGA parameters (*H*_1_, *H*_2_, *H*_3_, *H*_3_/*H*_2_, *WL*_1_, *WL*_2_, *WL*_3_, *Res* and *WL*_3_/*WL*_2_) of HA-L and FA-L extracts from the compost and non-composted organic fertilizer (highlighted); Table S8. Carbon content of the extracts HA-L, FA-L and DOM and yield (Y) of the extractions of HA-L and FA-L. The carbon content of each extract was calculated using Equations S1 and S2.; Table S9. Abundance of deprotonated groups at pH 7.0 (Q_pH7.0_) and the abundance of acid sites of the extracts HA-L, FA-L and DOM (*M_T_*, *M_T_*_,*HS*_, and *M_T_*_,*DOM*_). The abundance of acid sites of the extracts was calculated using Equations S3 and S4.; Table S10. Binding capacities (*c_ML_*_,*L*_, *c_ML_*_,*H*_) of the extracts HA-L, FA-L, and DOM.; Table S11. Characterization results of the compost samples and non-composted organic fertilizer (highlighted).; Table S12. TGA and DSC parameters (*WL*_1_, *WL*_2_, *WL*_3_, *WL*_4_, *Res*, *WL*_3_/*WL*_2_, *H*_1_, *H*_2_, *H*_3_) of the compost samples and non-composted organic fertilizer (highlighted).; Table S13: Definitions of the parameters used in the statistical analysis.

## References

[1] Donn S., Wheatley R.E., McKenzie B.M., Loades K.W., Hallett P.D. (2014). Improved soil fertility from compost amendment increases root growth and reinforcement of surface soil on slopes. Ecol. Eng..

[2] Guo X., Liu H., Wu S. (2019). Humic substances developed during organic waste composting: Formation mechanisms, structural properties, and agronomic functions. Sci. Total Environ..

[3] Bertoldi M., Sequi P., Lemmes B., Papi T. (1996). The Science of Composting.

[4] Spaccini R., Piccolo A. (2009). Molecular characteristics of humic acids extracted from compost at increasing maturity stages. Soil Biol. Biochem..

[5] Silva A.C., Rocha P., Antelo J., Valderrama P., López R., Geraldo D., Proença M.F., Pinheiro J.P., Fiol S., Bento F. (2022). Comparison of a variety of physico-chemical techniques in the chronological characterization of a compost from municipal wastes. Process Saf. Environ. Prot..

[6] El Ouaqoudi F.Z., El Fels L., Winterton P., Lemée L., Amblès A., Hafidi M. (2014). Study of humic acids during composting of ligno-cellulose waste by infra-red spectroscopic and thermogravimetric/thermal differential analysis. Compost Sci. Util..

[7] Chang Chien S.W., Wang M.C., Huang C.C. (2006). Reactions of compost-derived humic substances with lead, copper, cadmium, and zinc. Chemosphere.

[8] Liu H., Wang L., Zhong R., Bao M., Guo H., Xie Z. (2022). Binding characteristics of humic substances with Cu and Zn in response to inorganic mineral additives during swine manure composting. J. Environ. Manag..

[9] Silva A.C., Rocha P., Geraldo D., Cunha A., Antelo J., Pinheiro J.P., Fiol S., Bento F. (2023). Developing a compost quality index (CQI) based on the electrochemical quantification of Cd (HA) reactivity. Molecules.

[10] Muniz D.H.F., Oliveira-Filho E.C. (2023). Multivariate statistical analysis for water quality assessment: A review of research published between 2001 and 2020. Hydrology.

[11] Balabanova B., Stafilov T., Šajn R. (2021). use of multivariate statistical techniques to determine the source apportionment of heavy metals in soils and sediments. Heavy Metals in the Environment.

[12] Shrestha S., Kazama F. (2007). Assessment of surface water quality using multivariate statistical techniques: A case study of the Fuji river basin, Japan. Environ. Model. Softw..

[13] Gad M., Khomami N.T.S., Krieg R., Schor J., Philippe A., Lechtenfeld O.J. (2025). Environmental drivers of dissolved organic matter composition across central european aquatic systems: A novel correlation-based machine learning and FT-ICR MS approach. Water Res..

[14] Tassano M., Montañez A., Nuñez L., Trasante T., González J., Irigoyen J., Cabral P., Cabrera M. (2021). Spatial cross-correlation between physicochemical and microbiological variables at superficial soil with different levels of degradation. CATENA.

[15] Zhang J., Feng Y., Wu M., Chen R., Li Z., Lin X., Zhu Y., Delgado-Baquerizo M. (2021). Evaluation of microbe-driven soil organic matter quantity and quality by thermodynamic theory. mBio.

[16] Lasaridi K., Protopapa I., Kotsou M., Pilidis G., Manios T., Kyriacou A. (2006). Quality assessment of composts in the Greek market: The need for standards and quality assurance. J. Environ. Manag..

[17] Kucbel M., Raclavská H., Růžičková J., Švédová B., Sassmanová V., Drozdová J., Raclavský K., Juchelková D. (2019). Properties of composts from household food waste produced in automatic composters. J. Environ. Manag..

[18] Chin Y.-P., Aiken G., O’Loughlin E. (1994). Molecular weight, polydispersity, and spectroscopic properties of aquatic humic substances. Environ. Sci. Technol..

[19] Jamroz E., Bekier J., Medynska-Juraszek A., Kaluza-Haladyn A., Cwielag-Piasecka I., Bednik M. (2020). The contribution of water extractable forms of plant nutrients to evaluate MSW compost maturity: A case study. Sci. Rep..

[20] Giovanela M., Crespo J.S., Antunes M., Adamatti D.S., Fernandes A.N., Barison A., Da Silva C.W.P., Guégan R., Motelica-Heino M., Sierra M.M.D. (2010). Chemical and spectroscopic characterization of humic acids extracted from the bottom sediments of a Brazilian subtropical microbasin. J. Mol. Struct..

[21] See J.H., Bronk D.A. (2005). Changes in C:N ratios and chemical structures of estuarine humic substances during aging. Mar. Chem..

[22] Fuentes M., Baigorri R., González-Gaitano G., García-Mina J.M. (2007). The complementary use of 1H NMR, 13C NMR, FTIR and size exclusion chromatography to investigate the principal structural changes associated with composting of organic materials with diverse origin. Org. Geochem..

[23] Pertusatti J., Prado A.G.S. (2007). Buffer capacity of humic acid: Thermodynamic approach. J. Colloid Interface Sci..

[24] Amir S., Jouraiphy A., Meddich A., El Gharous M., Winterton P., Hafidi M. (2010). Structural study of humic acids during composting of activated sludge-green waste: Elemental analysis, FTIR and 13C NMR. J. Hazard. Mater..

[25] Ait Baddi G., Hafidi M., Cegarra J., Alburquerque J.A., Gonzálvez J., Gilard V., Revel J.-C. (2004). Characterization of fulvic acids by elemental and spectroscopic (FTIR and 13C-NMR) analyses during composting of olive mill wastes plus straw. Bioresour. Technol..

[26] Silva M.E.F., Lemos L.T., Bastos M.M.S.M., Nunes O.C., Cunha-Queda A.C. (2013). Recovery of humic-like susbtances from low quality composts. Bioresour. Technol..

[27] Hanc A., Enev V., Hrebeckova T., Klucakova M., Pekar M. (2019). Characterization of humic acids in a continuous-feeding vermicomposting system with horse manure. Waste Manag..

[28] González Pérez M., Martin-Neto L., Saab S.C., Novotny E.H., Milori D.M.B.P., Bagnato V.S., Colnago L.A., Melo W.J., Knicker H. (2004). Characterization of humic acids from a brazilian oxisol under different tillage systems by EPR, 13C NMR, FTIR and fluorescence spectroscopy. Geoderma.

[29] López R., Antelo J., Silva A.C., Bento F., Fiol S. (2021). Factors That affect physicochemical and acid-base properties of compost and vermicompost and its potential use as a soil amendment. J. Environ. Manag..

[30] Silva A.C., Teixeira A., Antelo J., Valderrama P., Oliveira R., Cunha A., Gley R., Pinheiro J.P., Fiol S., Bento F. (2022). Distinctive features of composts of different origin: A thorough examination of the characterization results. Sustainability.

[31] Zhao S., Guo Y., Sheng Q., Shyr Y. (2014). Advanced heat map and clustering analysis using heatmap3. BioMed Res. Int..

[32] Leenheer J.A. (1981). Comprehensive approach to preparative isolation and fractionation of dissolved organic carbon from natural waters and wastewaters. Environ. Sci. Technol..

[33] Smidt E., Tintner J. (2007). Application of differential scanning calorimetry (DSC) to evaluate the quality of compost organic matter. Thermochim. Acta.

[34] Díaz M.J., Ruiz-Montoya M., Palma A., de-Paz M.-V. (2021). Thermogravimetry Applicability in compost and composting research: A review. Appl. Sci..

