# Supplementary material for: A Correlation-Based Approach for Predicting Humic Substance Bioactivity from Direct Compost Characterization
Source: Molecules. 2025 Mar 28;30(7):1511. doi: 10.3390/molecules30071511 (PMC11990310; doi:10.3390/molecules30071511)
Supplement: Supplementary file 1 [file molecules-30-01511-s001.zip › molecules-3513829-supplementary.pdf]

Supplementary Information

Table S1. Identification of the compost and fertilizer samples characterized in the present work.

Sample	Composting methodology	Raw material
Compost of urban waste **(CUW)**	Tunnel composting	Selective collection of household food waste, food waste from restaurants, canteen, markets, fairs, festivities, pilgrimages and events, green waste from cemetery and household
Vermicompost of algae **(CVA)**	Vermicomposting	60% animal waste and 40% vegetable remains (fruits and algae), digested by *Eisenia foetida* earthworms.
Vermicompost of domestic waste **(CVDW)**		Green waste (flowers, leaves, grass, fruit peels) and brown waste (straw, dry leaves, dry grass), digested by *Eisenia foetida* or *Lumbricus rubellus* earthworms.
Compost of livestock waste **(CLW)**	Pile composting	100% animal waste (a mixture of 5% sheep manure without straw, 25% chicken manure and 70% pig manure)
Compost of algae **(CA)**		60% animal waste and 40% vegetable remains (fruits and algae).
Compost of sewage sludge **(CSS)**		Forestry waste, sludge from urban wastewater treatment and sludge from local effluent treatment.
Domestic compost of domestic waste **(CDDW)**	Domestic Composting	Green waste (flowers, leaves, grass, fruit peels) and brown waste (straw, dry leaves, dry grass).
**Organic Fertilizer**		
Fertilizer of livestock waste **(FLW)**	-	100% animal waste (chicken manure). Mixture subjected to a high temperature to eliminate pathogens.

Table S2. Characterization results of equilibrium solutions from the compost and non-composted organic fertilizer (highlighted).

Parameter	CVA	CVDW	CA	CLW	CUW	CDDW	CSS	FLW
**DOC (mg dm^{-3})**	111	717	75.23	861	548	115	698	921
**C_{oxi} (%)**	0.81	4.33	2.66	9.69	3.73	1.22	4.53	11.9
**Cl^- (mg dm^{-3})**	218	63.2	107	977	392	39.2	75.0	183
**F^- (mg dm^{-3})**	0.04	0.03	0.07	0.12	0.18	0.17	0.12	0.11
**SO_4^{2-} (mg dm^{-3})**	41.7	0.03	157	344	105	7.91	362	249
**PO_4^{3-} (mg dm^{-3})**	241	70.3	12	124	29.5	45.2	29.0	189
**NO_2^- (mg dm^{-3})**	1.8	3.9	0.54	6.7	3.9	11.0	2.2	9.17
**NO_3^- (mg dm^{-3})**	609	0.2	40.55	2.6	5.2	203	5.0	0.48
**NH_4^+ (mg dm^{-3})**	14.1	11.1	1.77	21.2	7.8	4.6	159	16.3
**NH_4^+/NO_3^-**	0.02	69.5	0.04	8.31	1.49	0.02	31.9	34.0
**Si (mg dm^{-3})**	6.2	2.6	1.10	19	1.4	4.1	1.6	8.03
**Ca (mg dm^{-3})**	80.7	17.8	11.4	22.5	28.0	28.6	95.7	32.4
**Mg (mg dm^{-3})**	66.1	15.1	17.1	38.9	8.8	12.3	55.8	35.0
**Na (mg dm^{-3})**	114	26.7	72.1	552	175	17.3	21.1	115
**K (mg dm^{-3})**	431	333	103.7	1364	349	150	55	724
**Fe (mg dm^{-3})**	0.01	0.23	0.1	7.00	1.09	0.05	19.3	8.18
**Al (mg dm^{-3})**	< DL	0.1	< DL	0.2	0.3	< DL	0.3	0.08
**As (µg dm^{-3})**	24	13	24.2	57	54	23	42	18
**Cu (mg dm^{-3})**	0.03	0.09	< DL	1.40	0.11	0.02	0.21	0.34
**Mn (mg dm^{-3})**	0.48	0.02	< DL	0.16	0.03	< DL	0.33	0.46

Note: < DL (lower than the detection limit)

Table S3. UV-vis parameters (ε_{280}, aromaticity and molar mass) of DOM, HA-L and FA-L from the compost and non-composted organic fertilizer (highlighted).

Parameter		CVA	CVDW	CA	CLW	CUW	CDDW	CSS	FLW
ε_{280} (L molC^{-1} cm^{-1})	HA-L	381	347	408	323	513	145	451	289
	FA-L	381	243	407	247	419	117	351	209
	DOM	306	291	366	267	280	402	146	491
Aromaticity (%)	HA-L	25.8	24.1	27.2	22.9	32.4	29.3	14.0	21.2
	FA-L	25.8	18.9	27.1	19.1	27.7	24.3	12.6	17.2
	DOM	22.1	21.3	25.0	20.1	20.7	26.9	14.0	31.3
Molar mass (g mol^{-1})	HA-L	2011	1875	2119	1780	2538	2290	1069	1644
	FA-L	2011	1460	2114	1476	2163	1891	958	1324
	DOM	1712	1651	1949	1555	1605	2095	1072	2451

Note: *molC* refers to moles of carbon, indicating that the extinction coefficient is normalized to the carbon content of the dissolved organic matter.

Table S4. Physicochemical characterization parameters (C, N, H, S and O) and yield (Y) of the extractions of HA-L and FA-L from the compost and non-composted organic fertilizer (highlighted). The values have been rectified on the basis of ash content.

Parameter		CVA	CVDW	CA	CLW	CUW	CDDW	CSS	FLW
**C (wt%)**	HA-L	53.5	52.7	53.3	55.2	54.5	50.6	58.5	57.5
	FA-L	49.7	39.8	45.8	35.4	40.8	38.4	40.9	36.9
**N (wt%)**	HA-L	3.71	6.12	6.02	5.62	6.74	4.92	5.79	7.24
	FA-L	3.72	3.20	4.13	3.36	4.37	2.62	6.05	5.72
**H (wt%)**	HA-L	5.46	4.65	5.68	5.44	6.14	4.47	8.28	8.31
	FA-L	5.36	3.61	4.61	3.36	6.02	3.09	5.83	4.32
**S (wt%)**	HA-L	1.14	0.41	1.61	2.07	0.91	0.50	1.88	1.08
	FA-L	1.08	0.46	2.16	1.80	0.96	0.41	2.22	1.11
**O (wt%)**	HA-L	36.2	36.1	33.4	31.7	31.7	39.5	25.5	25.8
	FA-L	40.1	52.9	43.3	56.1	47.8	55.5	45.0	51.91
**Ash (wt%)**	HA-L	1.00	1.70	0.76	12.67	8.40	6.48	1.39	5.80
	FA-L	11.82	9.17	42.19	10.97	4.27	13.73	21.57	7.70
**Yield (g kg^{-1})**	HA-L	33.4	26.4	19.9	34.9	21.1	23.8	19.0	53.6
	FA-L	2.3	4.7	2.7	7.0	3.3	4.8	4.2	10.9

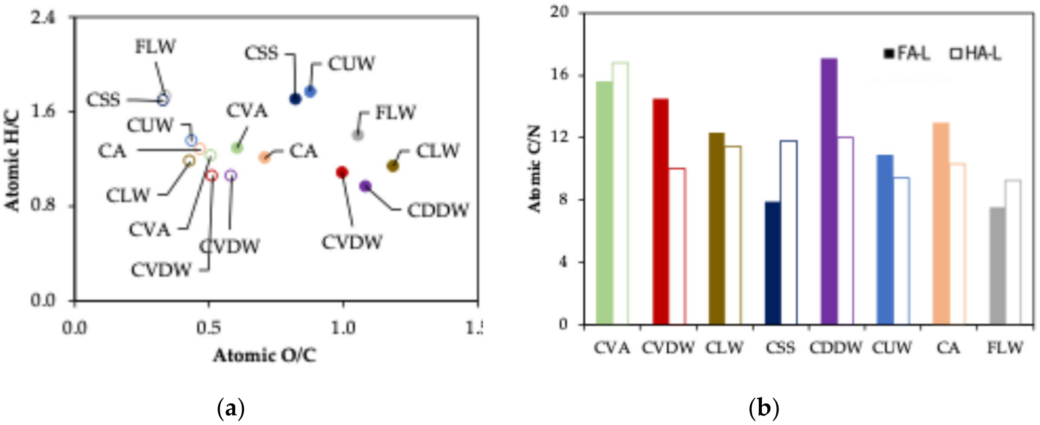

Figure S1. (a) Van Krevelen diagram (O/C *vs.* H/C atomic ratios) and **(b)** C/N atomic ratio of (o) HA-L and (●) FA-L extracts of the samples: CDDW (purple), CVDW (red), CVA (green), CA (orange), CUW (blue), CSS (dark blue), CLW (brown) and FLW (gray).

Table S5. ^{1}H-NMR parameters (Aliphatic protons, Aromatic protons and H_{aro}/H_{ali}) of FA-L and HA-L from the compost and non-composted organic fertilizer (highlighted).

Chemical shifts intervals	δ 0.4 – 4.5 ppm		δ 6 - 9 ppm		Ratio Aromatic/Aliphatic protons (H_{aro}/H_{ali})	
Functional groups	Aliphatic protons		Aromatic protons			
Samples	**FA-L**	**HA-L**	**FA-L**	**HA-L**	**FA-L**	**HA-L**
**CVA**	7.49	13.7	1.43	3.45	0.191	0.252
**CVDW**	8.93	17.6	0.95	2.26	0.106	0.129
**CLW**	9.95	16.0	1.46	2.69	0.147	0.168
**CSS**	13.6	27.9	0.77	2.04	0.056	0.073
**CDDW**	8.20	16.2	1.07	2.10	0.130	0.130
**CUW**	5.09	13.4	0.44	1.80	0.086	0.134
**CA**	2.23	13.7	0.46	1.91	0.206	0.139
**FLW**	10.2	19.3	1.5	3.11	0.147	0.161

Table S6. ATR-FTIR parameters (***$I_{1630/2845}$*** and ***$I_{1630/2925}$***) of HA-L and FA-L from the compost and non-composted organic fertilizer (highlighted).

**Parameter**		**CVA**	**CVDW**	**CA**	**CLW**	**CUW**	**CDDW**	**CSS**	**FLW**
$I_{1630/2845}$	HA-L	2.42	4.45	5.23	3.61	4.31	4.01	2.63	2.42
	FA-L	4.17	2.67	3.47	5.28	9.08	3.52	4.32	n.d.
$I_{1630/2925}$	HA-L	3.46	5.01	8.01	3.46	4.70	4.91	1.76	3.46
	FA-L	4.02	2.61	3.13	3.88	7.56	2.59	3.17	3.43

Note: n.d. (not detected)

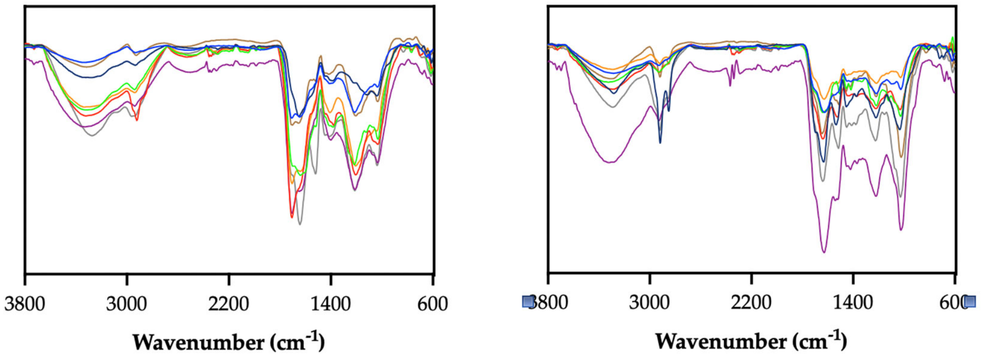

(**a**) (**b**)

Figure S2. ATR-FTIR spectra of (**a**) FA-L and of (**b**) HA-L obtained from the samples: CDDW (purple), CVDW (red), CVA (green), CA (orange), CUW (blue), CSS (dark blue), CLW (brown) and FLW (gray).

Table S7. DSC and TGA parameters (*H1*, *H2*, *H3*, *H3*/*H2*, *WL1*, *WL2*, *WL3*, *Res* and *WL3*/*WL2*) of HA-L and FA-L extracts from the compost and non-composted organic fertilizer (highlighted).

Parameter		CVA	CVDW	CA	CLW	CUW	CDDW	CSS	FLW
H_1 (kJ g^{-1})	HA-L	0.39 ± 0.09	0.14 ± 0.03	0.37 ± 0.09	0.07 ± 0.02	0.27 ± 0.06	0.18 ± 0.04	0.40 ± 0.09	0.07 ± 0.02
	FA-L	0.25 ± 0.06	0.07 ± 0.02	0.20 ± 0.05	0.05 ± 0.01	0.47 ± 0.11	0.23 ± 0.05	0.03 ± 0.01	0.07 ± 0.02
H_2 (kJ g^{-1})	HA-L	-0.82 ± 0.11	-0.67 ± 0.09	-1.10± 0.14	-0.66 ± 0.09	-1.3 ± 0.2	-0.17 ± 0.02	-1.4 ± 0.2	-1.8 ± 0.2
	FA-L	-3.6 ± 0.5	-3.4 ± 0.4	-1.54 ± 0.20	-1.7 ± 0.2	-3.1 ± 0.4	-0.57 ± 0.07	-1.2 ± 0.2	-2.1± 0.3
H_3 (kJ g^{-1})	HA-L	-1.2± 0.06	-1.0± 0.05	-1.09± 0.05	-0.52 ± 0.02	-4.5 ± 0.2	-2.8 ± 0.1	-1.2 ± 0.06	-4.7 ± 0.2
	FA-L	-3.7± 0.2	-2.2 ± 0.1	-1.20 ± 0.06	-1.6 ± 0.08	-2.5 ± 0.1	-2.6 ± 0.1	-2.7± 0.1	-3.2± 0.2
H_3/H_2	HA-L	1.5 ± 0.2	1.5 ± 0.2	0.99 ± 0.1	0.79 ± 0.1	3.6 ± 0.5	-16 ± 2.2	0.8 ± 0.1	2.6 ± 0.4
	FA-L	1.0 ± 0.1	3.6 ± 0.5	0.78 ± 0.1	0.94 ± 0.1	0.82 ± 0.1	4.5 ± 0.6	2.3 ± 0.3	1.5 ± 0.2
WL_1 (%)	HA-L	3.9 ± 1.0	7.1 ± 1.8	5.0 ± 1.3	7.5 ± 2.0	8.3 ± 2.2	8.6 ± 2.2	2.4 ± 0.6	2.3 ± 0.6
	FA-L	4.5 ± 1.2	3.3 ± 0.9	1.4 ± 0.4	3.4 ± 0.9	6.4 ± 1.7	4.1 ± 1.1	2.4 ± 0.6	4.5 ± 1.2
WL_2 (%)	HA-L	34.5 ± 2.7	32.9 ± 2.6	32.7 ± 2.6	30.5 ± 2.4	30.9 ± 2.4	30.9 ± 2.4	43.1 ± 3.4	38.7 ± 3.0
	FA-L	42.5 ± 3.3	35.6 ± 2.8	31.2 ± 2.4	20.2 ± 1.6	38.4 ± 3.0	31.1 ± 2.4	29.1 ± 2.3	31.2 ± 2.4
WL_3 (%)	HA-L	49.3 ± 4.3	46.8 ± 4.1	52.6 ± 4.6	50.4 ± 4.4	58.7 ± 5.2	54.4 ± 4.8	21.4 ± 1.9	54.5 ± 4.8
	FA-L	37.5 ± 3.3	35.6 ± 3.1	27.2 ± 2.4	41.2 ± 3.6	33.5 ± 2.9	34.1 ± 3.0	31.7 ± 2.8	37.4 ± 3.3
Res (%)	HA-L	16.6 ± 1.1	12.3 ± 0.8	8.40 ± 0.5	11.8 ± 0.8	2.50 ± 0.2	4.7 ± 0.3	32.1 ± 2.1	4.5± 0.3
	FA-L	12.7 ± 0.8	23.1 ± 1.5	40.2 ± 2.6	33.4 ± 2.2	21.6 ± 1.4	29 ± 1.9	35.7 ± 2.3	26.8 ± 1.7
WL_3/WL_2	HA-L	1.4 ± 0.2	1.4 ± 0.2	1.6 ± 0.2	1.7 ± 0.2	1.9 ± 0.2	1.8 ± 0.2	0.5 ± 0.06	1.4 ± 0.2
	FA-L	0.9 ± 0.1	1.0 ± 0.1	0.87 ± 0.10	2.0 ± 0.2	0.9 ± 0.1	1.1 ± 0.1	1.1 ± 0.1	1.2 ± 0.1

Data obtained through acid-base titrations following the methodology proposed by López et al. (2021) [30].

The carbon content of each extract C_{HS} and C_{DOM} were calculated using equations (S1) and (S2), for the HS and DOM, respectively:

$$C_{HS} = \frac{C}{100} \times Y \quad \text{(S1)}$$

$$C_{DOM} = \frac{DOC}{\frac{m_{compost}}{V}} \quad \text{(S2)}$$

where C is the carbon content of the HS (% w/w), Y is the yield of the extractions (w/w, g kg^{-1}), DOC is the concentration of dissolved organic carbon (mg L^{-1}) and $m_{compost}/V$ is the mass of compost *per* volume of solution used to prepared the equilibrium solution (50 g$_{compost}$ L^{-1}).

Table S8. Carbon content of the extracts HA-L, FA-L and DOM and yield (Y) of the extractions of HA-L and FA-L. The carbon content of each extract was calculated using Equations S1 and S2.

Sample	HA-L			FA-L			DOM	
	C (%)	Y (g kg^{-1})	C_{HA} (g_C $kg_{compost}^{-1}$)	C (%)	Y (g kg^{-1})	C_{FA} (g_C $kg_{compost}^{-1}$)	DOC (mg L^{-1})	C_{DOM} (g_C $kg_{compost}^{-1}$)
**CVA**	53.5*	33.4*	17.9	49.7*	2.27*	1.14	111*	2.22
**CVDW**	52.7*	19.0*	13.9	39.8*	4.73*	1.87	717*	14.3
**CA**	53.3	19.9*	10.6	45.8*	2.67*	1.22	75.2*	1.5
**CLW**	55.2*	34.9*	19.3	35.4*	7.00*	2.48	861*	17.2
**CUW**	54.5*	21.1*	11.5	40.8*	3.33*	1.35	548*	11
**CDDW**	50.6*	26.4*	9.62	38.4*	4.20*	1.61	115*	2.31
**CSS**	58.5*	23.8*	13.9	40.9*	4.84*	1.97	698*	14
**FLW**	57.5	53.6	30.9	36.9*	10.9*	4.02	921*	18.4

* Data from López et al. (2021) [30].

The abundance of the acid sites, $M_{T,HS}$ and $M_{T,DOM}$ (expressed in mol $kg_{compost}^{-1}$) were calculated attending to the values of M_T from each extract (Table S9, expressed in mmol g_C^{-1}) and to C_{HS} or C_{DOM} (Table S8, expressed in g_C $kg_{compost}^{-1}$), using equations (S3) and (S4).

$$M_{T,HS} = M_T \times C_{\mathrm{HS}} \quad \text{(S3)}$$

$$M_{T,DOM} = M_T \times C_{DOM} \quad \text{(S4)}$$

Table S9. Abundance of deprotonated groups at pH 7.0 ($Q_{pH7.0}$) and the abundance of acid sites of the extracts HA-L, FA-L and DOM (M_T, $M_{T,HS}$ and $M_{T,DOM}$). The abundance of acid sites of the extracts was calculated using Equations S3 and S4.

	Parameters		CVA*	CVDW*	CA	CLW*	CUW*	CDDW*	CSS*	FLW
HA-L	$Q_{pH\ 7.0}$	(mmol g_C^{-1})	4.18	4.53	4.92	4.23	5.39	5.43	3.57	3.74
	M_T	(mmol g_C^{-1})	6.65	7.13	8.28	7.14	8.95	8.73	6.32	5.68
	$M_{T,HS}$	(mol $kg_{compost}^{-1}$)	0.12	0.1	0.09	0.14	0.1	0.08	0.09	0.18
FA-L	$Q_{pH\ 7.0}$	(mmol g_C^{-1})	8.10	10.2	10.9	8.46	9.12	9.41	11.0	7.09
	M_T	(mmol g_C^{-1})	11.3	14.1	16.6	12.9	12.5	13.7	18.5	10.6
	$M_{T,HS}$	(mol $kg_{compost}^{-1}$)	0.01	0.03	0.02	0.03	0.02	0.02	0.04	0.04
DOM	$Q_{pH\ 7.0}$	(mmol g_C^{-1})	4.59	2.18	3.31	2.36	1.85	5.65	2.84	2.55
	M_T	(mmol g_C^{-1})	9.89	4.32	6.7	5.63	3.92	11.3	8.38	5.92
	$M_{T,DOM}$	(mol $kg_{compost}^{-1}$)	0.22	0.62	0.10	0.97	0.43	0.26	1.17	1.09

* Data from López et al. (2021) [30].

Table S10. Binding capacities ($c_{ML,L}$, $c_{ML,H}$) of the extracts HA-L, FA-L and DOM.

		Parameters	CVA*	CVDW*	CA	CLW*	CUW*	CDDW*	CSS*	FLW
HA-L	$c_{ML,L}$	**(L mg^{-1} compost)**	24.4	43	276.2	30.7	67.5	36.7	17.1	104
	$c_{ML,H}$	**(L mg^{-1} compost)**	5.0	36.7	65.8	14.3	13.3	16.5	4.3	65.8
FA-L	$c_{ML,L}$	**(L mg^{-1} compost)**	1.1	1.6	3.5	5.5	4.1	2.4	1.9	5.6
	$c_{ML,H}$	**(L mg^{-1} compost)**	0.5	0.8	1.2	1.8	0.8	0.5	1.4	4.6
DOM	$c_{ML,L}$	**(L kg^{-1} compost)**	0.9	9.1	1.5	86.1	29.2	2.1	4.7	1.49
	$c_{ML,H}$	**(L kg^{-1} compost)**	0.4	2.1	0.5	18	14.9	0.6	1.5	0.51

* Data from Silva et al. (2022, 2023) [5,9,30]

Table S11. Characterization results of the compost samples and non-composted organic fertilizer (highlighted).

Parameter	CVA	CVDW	CLW	CSS	CDDW	CUW	CA	FLW
C (%)	29.7	43.9	22.8	31.3	23.3	34.0	6.0	22.5
C_{oxi} (%)	27.5	33.1	17.8	30.6	18.8	29.3	5.7	15.4
N (%)	2.4	4.8	1.8	3.6	2.3	2.7	0.6	1.7
S (%)	0.4	0.4	0.7	1.1	0.2	0.2	0.23	0.1
C/N	12.5	9.2	12.8	8.6	10.3	12.6	9.97	13.2
C_{oxi}/C	92.5	75.4	77.8	97.8	81.0	86.1	94.6	68.2
Ca (g kg^{-1})	13.1	9.6	78.0	19.9	16.1	77.5	11.8	212
Mg (g kg^{-1})	4.5	4.4	18.2	5.2	3.6	3.7	3.1	6.2
Na (g kg^{-1})	1.8	1.1	10.5	2.2	0.6	4.8	1.5	3.0
K (g kg^{-1})	10.2	14.6	30.2	4.3	7.6	12.2	4.7	18.3
Fe (g kg^{-1})	3.47	0.88	6.46	23.1	13.0	4.02	4.40	1.70
Al (g kg^{-1})	3.11	1.28	8.26	24.7	19.9	5.71	6.11	2.40
As (mg kg^{-1})	3.3	n.d.	8.8	8.2	3.8	6.3	n.d.	3.2
Cu (g kg^{-1})	0.05	0.05	0.18	0.25	0.07	0.04	0.03	0.07
Mn (g kg^{-1})	0.44	0.07	0.35	0.25	0.18	0.13	0.15	0.29

Note: n.d. (not detected)

* Data from López et al. (2021) [30] and Silva et al. (2022) [5,32]

Table S12. TGA and DSC parameters (*WL_1, WL_2, WL_3, WL_4, Res, WL_3/WL_2, H_1, H_2, H_3*) of the compost samples and non-composted organic fertilizer (highlighted).

Parameter	CVA	CVDW	CLW	CSS	CDDW	CUW	CA	FLW
***WL_1* (%)**	7.6 ± 2.0	12.5 ± 3.2	9.3 ± 2.4	13.2 ± 3.4	8.6 ± 2.2	9.3 ± 2.4	2.0 ± 0.5	7 ± 1.8
***WL_2* (%)**	31.3 ± 2.4	35.2 ± 2.7	20.5 ± 1.6	31.9 ± 2.5	23.0 ± 1.8	28.1 ± 2.2	6.1 ± 0.47	21.2 ± 1.6
***WL_3* (%)**	23.2 ± 2.0	27.4 ± 2.4	19.3 ± 1.7	18.7 ± 1.6	22.0 ± 1.9	27.0 ± 2.4	5.0 ± 0.68	16.7± 2.3
***WL_4* (%)**	0.4 ± 0.1	17.2 ± 5.2	1.8 ± 0.5	0.2 ± 0.1	1.1 ± 0.3	7.4 ± 2.2	0.2 ± 0.06	13.8 ± 4.2
***Res* (%)**	37.3 ± 2.4	7.9 ± 0.5	48.9 ± 3.2	36.8 ± 2.4	46.3 ± 3.0	27.9 ± 1.8	88.2 ± 5.8	41.3 ± 2.7
WL_3/WL_2	0.74 ± 0.09	0.78 ± 0.09	0.94 ± 0.11	0.58 ± 0.07	0.96 ± 0.11	0.96 ± 0.11	0.82 ± 0.10	0.79 ± 0.09
***H_1* ($kJ\ g^{-1}$)**	0.25 ± 0.06	0.38 ± 0.09	0.28 ± 0.06	0.31 ± 0.07	0.31 ± 0.07	0.16 ± 0.04	0.08 ± 0.02	0.26 ± 0.06
***H_2* ($kJ\ g^{-1}$)**	-1.74 ± 0.22	-0.73 ± 0.09	-1.48 ± 0.19	-2.89 ± 0.37	-1.55 ± 0.20	-2.08 ± 0.27	-0.24 ± 0.03	-0.81 ± 0.10
***H_3* ($kJ\ g^{-1}$)**	-3.77 ± 0.18	-5.96 ± 0.28	-2.23 ± 0.10	-2.74 ± 0.13	-2.98 ± 0.14	-4.65 ± 0.22	-0.35 ± 0.02	-2.63 ± 0.12

* Data from Silva et al. (2022) [5,32]

Table S13. Definitions of the parameters used in the statistical analysis.

Parameters	Definition
C/N	carbon to nitrogen ratio
O/C	oxygen to carbon ratio
H/C	hydrogen to carbon ratio
ε_{280}	molar absorptivity coefficient
$I_{1630/2925}$	aromaticity index
H_{aro}/H_{ali}	aromatic hydrogen to aliphatic hydrogen ratio
WL_1/TWL	related to dehydration and desorption processes
WL_2/TWL	related to the easily biodegradable aromatic structures
WL_3/TWL	related to the degradation of complex aromatic structures, such as lignin, complex aromatic structures, and humic substances
WL_3/WL_2	a measure of the relative amount of the thermally more stable fraction of organic matter compared to the least stable
H_1	ascribed to dehydration reactions
H_2	related to the decomposition of recalcitrant organic components
H_3	related to the decomposition of extra-recalcitrant organic components
M_1	abundance of the carboxylic groups
M_2	abundance of phenolic groups
K_1	protonation constant of carboxylic-type groups
K_2	protonation constant of phenolic-type groups
$c_{ML,L}$	extent of binding for the lowest concentration of added Cd^{2+} (10^{-8} mol L^{-1})
$c_{ML,H}$	extent of binding for the highest concentration of added Cd^{2+}(10^{-6} mol L^{-1})
Yield (Y)	extraction yield
DOM parameters	
M_1	abundance of carboxylic groups in amino acids
M_2	abundance of carboxylic groups in organic acids
M_3	abundance of phenolic hydroxyl groups
K_1	protonation constant of carboxylic groups in amino acids
K_2	protonation constant of carboxylic groups in organic acids
K_3	protonation constant of phenolic hydroxyl groups
Compost parameters	
DOC	dissolved organic carbon
C_{oxi}	oxidizable carbon
C_{oxi}/C	oxidizable carbon to carbon ratio
CEC	cation exchange capacity
CEC/C	cation exchange capacity to carbon ratio.
Res	TGA residue
EC	electric conductivity
WL_1	dehydration and desorption processes
WL_2	easily biodegradable aromatic structures
WL_3	degradation of complex aromatic structures, such as lignin, complex aromatic structures, and humic substances
